# Species interactions are key to spatiotemporal gene expression and multilayer formation in *Stenotrophomonas maltophilia* K279a dual species biofilms

**DOI:** 10.1016/j.bioflm.2026.100374

**Published:** 2026-06-16

**Authors:** Raphael Moll, Tim Hoffmann, Calvin Tu, Roland Thünauer, Wolfgang R. Streit, Ifey Alio

**Affiliations:** a Department of Microbiology and Biotechnology, University of Hamburg, Hamburg, Germany; b HPI, Leibniz Institute of Experimental Virology, Hamburg, Germany

**Keywords:** Multispecies biofilms, Antibiotic resistance, *Stenotrophomonas maltophilia*, *Pseudomonas aeruginosa*, *Staphylococcus aureus*, Lattice light sheet microscopy (LLSM), Biofilm architecture, Quorum sensing (QS), Iron transport, Drug targets

## Abstract

Pathogenic multispecies biofilms are major drivers in the persistence and virulence of bacterial infections, complicating treatment due to their pronounced antibiotic resistance. To better understand the temporal and spatial dynamics within these complex communities, we established dual-species biofilm models focusing on the pathogen *Stenotrophomonas maltophilia* in combination with *Pseudomonas aeruginosa* and *Staphylococcus aureus*. Using Lattice Light Sheet Microscopy (LLSM) and automated cell quantification, we demonstrated the dynamic growth and complex spatial organization of these dual-species biofilms. The study identified both shared and species-specific strategies of biofilm formation, ultimately underscoring the dynamic and adaptive nature of the *S. maltophilia* K279a biofilm architecture. A vertical zonation was a general and pronounced trait of *S. maltophilia* biofilms. Marker gene expression in *S. maltophilia* was generally heterogeneous across the biofilm layers but followed a clear time-dependent on/off pattern. Iron transport and cytochrome biosynthesis appear to be key traits involved in niche competition. Furthermore, *S. maltophilia* attenuated *P. aeruginosa* N-acyl-homoserine lactone (AHL) quorum sensing (QS)-regulated gene expression involved in surface colonization, outer membrane biogenesis, and cyclic di-GMP signaling. Thereby we identified key drivers involved in the *S. maltophilia* dual-species biofilm lifestyle, representing potential drug targets to combat multi-species and heterogeneous biofilm infections.

## 1 Introduction

Biofilms, complex microbial communities encased in an extracellular polymeric substance (EPS) matrix, are fundamental to the survival and persistence of microorganisms in diverse natural and host environments [[1], [2], [3]] While single-species biofilms have been extensively researched, the critical role of multispecies biofilms is becoming increasingly recognized. In these consortia, different microbes coexist, engaging in dynamic synergistic and competitive interactions that profoundly influence the biofilm's architecture, function, stability, and, crucially, its resistance to external stressors [4].

The complexity of these polymicrobial communities is highly relevant in clinical settings, particularly for persistent human diseases, such as chronic lung or skin infections in immunocompromised patients [[5], [6], [7]]. Biofilm formation on lung surfaces is a hallmark of chronic conditions, including cystic fibrosis (CF), pneumonia, and COPD exacerbations [8]. In this state, the microbial matrix shields the community from the host immune system and antibiotic intervention, leading to pronounced antibiotic resistance and necessitating alternative treatment strategies like biofilm-disrupting agents and early preventative measures.

In these chronic contexts, infections are typically polymicrobial, often involving a consortium of Gram-negative and Gram-positive bacteria, along with fungi, co-localizing within complex biofilms. Chronic respiratory infections in cystic fibrosis patients is mainly caused by opportunistic bacterial pathogens such as *Pseudomonas aeruginosa* (PA), Methicillin-resistant or Methicillin-Sensitive *Staphylococcus aureus*, Burkholderia species, *Stenotrophomonas maltophilia* (SM) and *Achromobacter xylosoxidans* [[9], [10], [11]]. Several studies have also reported co infections involving *P. aeruginosa, S. maltophilia* and *S. aureus* in CF patients thereby making treatment difficult [12].

These microbes are adept at causing persistent infection and disease by expressing a diverse array of virulence factors, with robust biofilm formation enhancing their survival in challenging host environments.

Recent findings highlight the intricate nature of dual-species interactions, which can encompass both beneficial and antagonistic effects [[13], [14], [15]]. For instance, studies suggest that *P. aeruginosa* can either suppress or enhance the biofilm formation and antibiotic tolerance of *S. aureus* in a co-culture, depending on the specific strain and context [16]. Furthermore, *P. aeruginosa* molecules contained in cell-free supernatants (e.g., HQNO, pyoverdine, pyochelin) can decrease the susceptibility of *S. aureus* to vancomycin [17], while its quorum-sensing signals (like 3-oxo-C12-homoserine-lactone) are known to modulate the virulence of co-inhabiting microbes like *Candida albicans* [18]. This inherent synergistic and protective effect of multispecies biofilms against antimicrobials profoundly complicates clinical treatment [19,20]

Our previous work has contributed to this understanding by demonstrating that the 3D-structure of single-species *S. maltophilia* biofilms is highly strain-specific [21] Extending these investigations to multispecies biofilms, we previously showed that *S. maltophilia* profoundly affects the physiology and gene expression of co-colonizing species such as *P. aeruginosa* and *S. aureus* (Alio & Moll, 2023).

Specifically, RNA sequencing revealed differential regulation of genes related to lactate metabolism, transport systems, respiration, and quorum sensing. High-resolution confocal microscopy further showed that *S. maltophilia* typically colonizes the basal layer of multispecies biofilms, and revealed distinct, strain-specific patterns, including stratified layering and strong interspecies competition for nutrients (Fe^2+^, Pi, and Mg^2+^), alongside a shift toward fermentative metabolism.

Most studies have primarily relied on RNA sequencing to investigate how bacterial species influence each other's gene expression in multispecies biofilms. To date, however, no study has systematically examined the spatial and temporal dynamics of bacterial gene expression in response to the presence of other species within mixed-species biofilms. Specifically, it remains unknown when and in which biofilm layers particular genes are differentially expressed in mixed-species biofilms compared with single-species biofilms.

Within this established framework, the present study addresses a critical, unresolved question: How is the spatial and temporal organization of gene expression orchestrated during the development of *S. maltophilia* in a dual-species biofilms? Our focus is on profiling genes related to antibiotic resistance, virulence, and respiration, and determining precisely how these expression patterns are shaped by the complex multispecies interactions.

## 2 Materials & methods

### 2.1 Bacterial strains, chemicals, and growth conditions

In Table 1 strains and plasmids used in this study are summarized. All strains were cultured in either LB-medium (10 g/L tryptone, 5 g/L yeast extract and 5 g/L NaCl) or 10% LB-medium at 37°C.

**Table 1:** Bacteria strains and plasmids.

Strains & Plasmids	Description	Reference/Source
*E. coli* DH5α	F- ɸ80dlacZΔM15 Δ(argF-lacZYA) U169 endA1 hsdR17 (rK-, mK-) supE44 thi-1 recA1 gyrA96 relA1	[22]
*E. coli* SM10ƛpir	thi thr leu tonA lacY supE recA::RP4-2-Tc:Mu Km λpir	[23]

*S. maltophilia* K279a	wildtype isolate	[24]
K279a_pBBR1MCS: smlt4401:mCherry	Reporter fusion	This study
K279a_pBBR1MCS: smlt4401:mScarlet	Reporter fusion	This study
K279a_pBBR5MCS: Propionate CoA:mCherry	Reporter fusion	This study


*S. maltophilia* SM454	wildtype isolate	[25]
SM454_pBBR1MCS:smlt2713:mCherry	Reporter fusion	This study
SM454_pBBR1MCS:smlt2713:mScarlet	Reporter fusion	This study
SM454_pBBR1MCS::TIVSS:mCherry	Reporter fusion	This study
SM454_pBBR1MCS:stmPr2:mCherry	Reporter fusion	This study
*S. maltophilia* SKK55	wildtype isolate	[21]
SKK55_pBBR1MCS:bla2:mCherry	Reporter fusion	This study
SKK55_pBBR1MCS:bla2:mScarlet	Reporter fusion	This study
SKK55_pBBR1MCS:mraZ:mCherry	Reporter fusion	This study
K279a_sfGFP_UHH01	miniTn7T, pc promotor, Gm^r^, sfGFP	This study
K279a_tdtomato_UHH02	miniTn7T, pc promotor, Gm^r^, tdTomato	This study
K279a_BFP_UHH06	miniTn7T, pc promotor, Gm^r^, tagBFP	This study


*S. aureus* SH1000	Wildtype isolate	[26]
SH1000_sfGFP_UKE01	PCM29-SarA –sfGFP	This Study
SH1000_AmCyan_UKE02	PCM29-SarA –AmCyan	This Study
SH1000_mCherry_UKE03	PCM29-SarA –mCherry	This Study

*Candida albicans* SC5314	Wildtype isolate	(Amanda M. Gillum, 1984)
SC5314_sfGFP_UHH11	Mutant expressing EGFP	Alio & Moll et al., 2023
*P. aeruginosa* PAO1	wildtype isolate	(Grace, 2022)
PAO1_sfGFP_UHH07	miniTn7T, pc promotor, Gm^r^, sfGFP	This study
PAO1_mCherry_UHH08	miniTn7T, pc promotor, Gm^r^, mCherry	This study
*P. aeruginosa* PAO1_pBBR5MCS:nirG:mCherry	Reporter fusion	This study

*P. aeruginosa* PAO1_pBBR5MCS::VgrG5:mCherry *S. maltophilia* sm454 Δ *smlt2713*	Reporter fusion Deletion mutant	This study This study
**Plasmids**		
pRK2013	KanR; RK2-derived helper plasmid carrying the tra and mob genes for mobilization of plasmids containing oriT	(HELINSKI, 1979)
pUX-BF13	Transmissible plasmid containing oriVR6K. It carries tns genes, which are necessary for miniTn7 transfer	[27]
pUC18T-miniTn7T-Gm^r^	FRT-flanked Gm^r^ marker was inserted within miniTn7 element, Amp^r^	[27]
pUC18T-miniTn7T-Gm^r^-Pc	constitutive pc promotor of the class III Integron of *Delftia acidovorans* inserted after the Gm^r^ cassette	This study
pUC18T-miniTn7T-Gm^r^-Pc-sfGFP	fluorescent gene s*fGFP* inserted after the pc promotor	This study
pUC18T-miniTn7T-Gm^r^-Pc-mCherry	fluorescent gene *mCherry* inserted after the pc promotor	This study
pUC18T-miniTn7T-Gm^r^-Pc-tdtomato	fluorescent gene *tdTomato* inserted after the pc promotor	This study
pUC18T-miniTn7T-Gm^r^-Pc-tagBFP	fluorescent gene *tagBFP* inserted after the pc promotor	This study
pCM29_SarA	shuttle vector for *S. aureus*, expression of fluorescent genes from the SarA-P1 promoter; carries chloramphenicol resistance Cm^r^, only functional in gram- positives	This study
pCM29_SarA_sfGFP	fluorescent gene *sfGFP* inserted after the SarA promotor	This study
pJ-mCherry	fluorescent gene *mCherry* inserted after the SarA promotor	This Study
pJ-AmCyan	fluorescent gene *AmCyan* inserted after the SarA promotor	This Study
pBBR1-MCS	Broad host range vector, low copy, Cm^r^	(Michael E. Kovach, 1995)
pBBR1-MCS:smlt4401:mNeonGreen	pBBR1-MCS with the promotor cyoA (smlt4401) fused to *mNeonGreen*	This Study
pBBR5MCS: Propionate CoA:mCherry	Reporter fusion	This study
pBBR1MCS:smlt2713:mCherry	Reporter fusion	This study
pBBR1MCS:smlt2713:mScarlet	Reporter fusion	This study
pBBR1-MCS::TIVSS:mCherry	pBBR1-MCS with the promotor TIVSS fused to mCherry	This Study
pBBR1MCS:stmPr2:mCherry	Reporter fusion	This study
pBBR1-MCS:bla2:mCherry	pBBR1-MCS with the promotor bla2 fused to mCherry	This Study
pBBR1MCS:bla2:mScarlet	Reporter fusion	This study
pBBR1MCS:mraZ:mCherry	Reporter fusion	This study

### 2.2 Molecular cloning of reporter fusion constructs

Reporter fusion constructs were generated using the broad-host-range vector pBBR1MCS. Initially, the vector backbone was digested with *Sac*I and *Xba*I (New England Biolabs) restriction enzymes. The promoter regions of interest were amplified via PCR and digested with the same enzymes prior to ligation into the linearized vector. To insert the fluorescent reporter gene, either mCherry or mScarlet, the intermediate construct containing the promoter of the respective genes was subsequently digested with *Xba*I and *BamH*I (New England Biolabs). The fluorescent gene fragment, prepared with compatible overhangs, was ligated downstream of the promoter sequence. Correct assembly of the constructs was verified via colony PCR using M13 universal primers, flanking the multiple cloning site. PCR products of the expected sizes were purified and sent for Sanger sequencing to confirm sequence integrity and correct orientation of the inserts.

The finalized constructs were introduced into *Escherichia coli* DH5α via heat shock transformation for propagation and storage. For mobilization into the target organism, constructs were also transformed into *E. coli* WM3064, a diaminopimelic acid (DAP)-auxotrophic donor strain. Biparental conjugation was carried out between *E. coli* WM3064 harboring the plasmid and the respective target strains (K279a, sm454, SKK55, or PAO1). Transconjugants were selected on agar plates supplemented with appropriate antibiotics. Successfully transformed recipient strains were used for downstream applications such as fluorescence microscopy or plate reader-based fluorescence measurements. For long-term storage, strains were mixed 1:1 with 86% (v/v) glycerol and stored at −70°C.

### 2.3 Molecular cloning, labelling bacterial strains with fluorescent reporter genes and construction deletion mutants

The strains *S. maltophilia* K279a and *P. aeruginosa* PAO1 used in this study were chromosomally tagged with mini Tn7T system following published protocols [[27], [28], [29]]. All plasmids used in this approach are available in NCBI under accession numbers: OQ253286 – OQ253289, OP566392, OP566393 and OP566395. *S. aureus* SH1000 was labelled with which provides constitutive expression of sfGFP. For multi-colour labelling, additional plasmids enabling in trans expression of mCherry and AmCyan were constructed. Codon optimized genes (Eurofins, Germany) were cloned into pCM29 by Gibson assembly using backbone primers pCM_sarA_fwd/pCM-sarA_rev and fluorophore-specific primer pairs. The resulting constructs (pJ-mCherry, pJ-AmCyan) carry fluorophore-encoding sequences under the control of the sarA promoter resulting in constitutive fluorophore production. Correct assembly was verified by sequencing. Plasmids were introduced into *S. aureus* SH1000 via electroporation. The deletion mutants in *smlt2713* and in the background of *S. maltophila* sm454 were constructed using published protocols [30,31] and *sacB* as a deletion marker The primers for the construction of the smlt2713 deletion and all other primers used in this work are listed in Table S1.

### 2.4 Cultivation of biofilms in μ-slides

To investigate the biofilm architecture, strains were grown in μ-slide 8 well (ibidi Treat, Cat.No: 80826, ibidi USA Inc., Fitchburg, Wisconsin). For inoculation, an overnight culture was adjusted to 4.0 x 10^7^ cells/ml in 10% LB medium. The μ-slides were inoculated with 350 μl per well and incubated for 24h or 48h at 37°C in 10% LB under static conditions without medium replacement.

### 2.5 Fluorescence imaging analysis of biofilms

Live cell imaging was done at the Advanced Light and Fluorescence Microscopy (ALFM) facility of the CSSB in Hamburg, Germany. Visualization of μ-slide biofilms was performed using a lattice light sheet microscope (LLS 7) (Carl Zeiss Microscopy GmbH, Jena, Germany) equipped with an illumination objective (13.3x, NA 0.44), a detection objective (44.83x, NA 1.0), and two sCMOS cameras (Hamamatsu ORCA Fusion). Two laser lines at 488 nm and 561 nm were used as light sources.

### 2.6 Image analysis

3D images and videos of the biofilms were constructed from Z-series images using the ZEN software (version 2.3, Carl Zeiss Microscopy GmbH, Jena, Germany). Biofilm architecture was analyzed at least at three different positions for each strain and one representative LLS image or video was chosen. The biofilm mass was calculated with the BiofilmQ software [32], and the spatiotemporal kymo-graphs were constructed with the BiofilmQ visualization toolbox.

### 2.7 Reporter fusion assay

To quantify the gene expression of reporter fusion constructs, a reporter fusion assay was performed in a microtiter plate reader. Overnight cultures of the *S. maltophilia* reporter strains were prepared and incubated at 37°C with shaking at 130 rpm. On the following day, the optical density at 600 nm (OD600) was adjusted to 0.05 for single-species biofilms, or to 0.1 when cultures were co-incubated with different biofilm supernatants in 10% LB medium. Each well of a 96-well flat-bottom plate (Thermo Fisher Scientific; Nunclon™ Delta) contained 200 μl of diluted culture. For strains treated with biofilm supernatants, the OD600 was adjusted to 0.1, and 100 μl of the diluted cell suspension was transferred to each well, followed by the addition of 100 μl of supernatant. This resulted in a final OD600 of 0.05, comparable to cultures without supernatant treatment.

Three biological replicates with six technical replicates each were performed. Plates were incubated under static conditions at 37°C for 24 h. Every hour, OD600 and the emission of the respective fluorescent reporter were measured. To control the autofluorescence of the supernatants, wells containing only supernatants were included, and their values were subtracted from the sample measurements. Data was visualized using Microsoft Excel.

The use of biofilm supernatants, rather than mixed cultures, was essential for normalization of reporter activity to OD600. In mixed cultures, OD600 reflects the combined biomass of all strains, making it impossible to attribute growth and fluorescence to the reporter strain alone. By working with supernatants, only the OD600 of the reporter strain needed to be measured, enabling reliable normalization of fluorescence intensity to cell density.

### 2.8 Data availability

Figures, movies, supplementary tables, and processed/raw BiofilmQ data generated for this manuscript have been deposited in the Universität Hamburg Research Data Repository.

Figures are available at https://doi.org/10.25592/uhhfdm.18758, movies at https://doi.org/10.25592/uhhfdm.18730, and supplementary tables at https://doi.org/10.25592/uhhfdm.18759.

The BiofilmQ raw data are available as follows: K279a_4401_mScarlet + PAO1_sfGFP, https://doi.org/10.25592/uhhfdm.18734; K279a_4401_mScarlet_control, https://doi.org/10.25592/uhhfdm.18736; sm454_2713_mScarlet + PAO1_sfGFP, https://doi.org/10.25592/uhhfdm.18738; sm454_2713_mScarlet_control, https://doi.org/10.25592/uhhfdm.18740; SKK55_Bla2_sfGFP_pre_Amp_addition, https://doi.org/10.25592/uhhfdm.18742; SKK55_Bla2_sfGFP_post_Amp_addition, https://doi.org/10.25592/uhhfdm.18744; SKK55_Bla2_sfGFP_pre_and_post_Amp_addition_control, https://doi.org/10.25592/uhhfdm.18746; SKK55_Bla2_sfGFP + Amp_addition_at_0h, https://doi.org/10.25592/uhhfdm.18748; and SKK55_Bla2_sfGFP + Amp_addition_at_0h_control, https://doi.org/10.25592/uhhfdm.18750.

Previously published transcriptome data sets used in this study (Alio & Moll, 2023) are available under accession PRJEB56214 in NCBI/ENA/DDBJ.

## 3 Results

Since biofilm formation is a time- and spatial-dependent process, we asked in this study what the main signals and drivers are that influence time-resolved dual-species biofilm formation and structure for *Stenotrophomonas maltophilia* K279a, alongside *Pseudomonas aeruginosa* PAO1 and *Staphylococcus aureus* SH1000. To address this question, we first established a time-resolved Lattice light sheet (LLS) microscopy pipeline for single-species biofilms, and in a second step, we transferred this technology to dual-species biofilms grown in a static system. The Lattice light sheet microscopy has its major advantage in minimal phototoxicity and photobleaching since only the focal plane is illuminated at a given time.

By merging high-resolution live-cell imaging with detailed and high-resolution quantitative and time-resolved cell localization and numbering analysis, we were able to capture the dynamics of biofilm formation with unprecedented spatiotemporal detail in combination with the identification of key parameters involved in this fascinating process.

### 3.1 Single-species biofilms: High-resolution imaging reveals species-specific architecture and antibiotic adaptation

As indicated above, we first monitored single-species biofilm development of *S. maltophilia* K279a, *S. aureus* SH1000 and *P. aeruginosa* PAO1 to validate our system. Lattice light sheet microscopy (LLSM) was utilized to observe the morphological changes and structural complexity of the biofilms for 24 h. High-resolution 3D timelapse imaging was performed, capturing volumetric datasets every 30 min for a time period of 24 h. This approach enabled dynamic visualization of biofilm development with superior spatiotemporal resolution (Movie 1).

Supplementary data related to this article can be found online at https://doi.org/10.1016/j.bioflm.2026.100374

The following are the Supplementary data related to this article:

Multimedia component 8

**Fig. 1:**
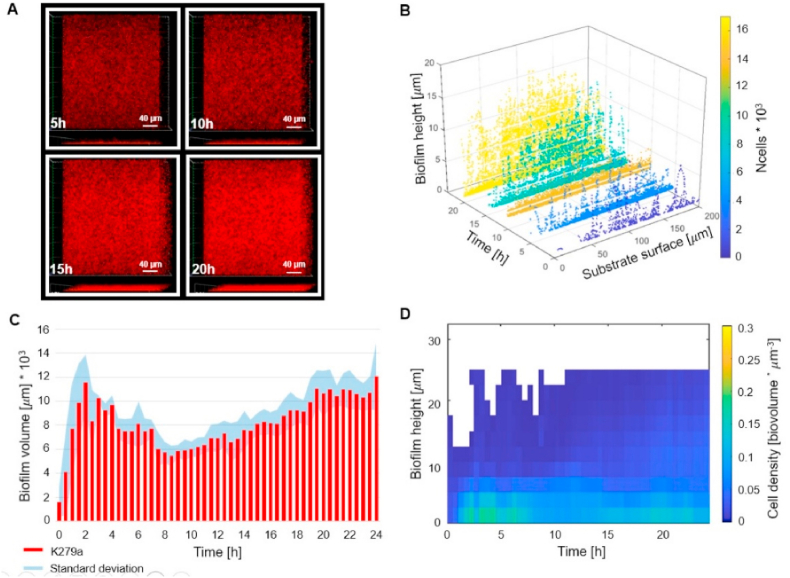
***Stenotrophomonas maltophilia* K279a forms structurally complex biofilms with dynamic spatial organization over 24 h.** Confocal laser scanning microscopy images illustrating the morphological changes of K279a biofilms at 5, 10, 15, and 20 h of growth. The images highlight the progressive maturation and structural complexity of the biofilms formed under static conditions in 10% LB at 37°C. **(B)** Quantification of cell distribution within a K279a biofilm at 5, 10, 15 and 20 h, indicating a shift in cell density and organization throughout the growth period. **(C)** The total biofilm volume of K279a measured from 0 to 24 h illustrates a dynamic growth pattern which demonstrates the robust characteristics of K279a. **(D)** Correlation of cell density to biofilm height of K279a from 0 to 24 h, revealing a direct relationship between cell density and biofilm architecture. Microscopy was performed using a lattice light sheet microscope, enabling high-resolution, volumetric imaging of biofilm architecture with minimal phototoxicity. Quantification was performed using the software BiofilmQ. Together, these results provide insight into the dynamic growth characteristics and spatial organization of K279a biofilms under the experimental conditions tested.

For *Stenotrophomonas maltophilia* K279a, we observed a dynamic process of biofilm maturation over a 24-h period. LLSM imaging revealed a progressive increase in structural complexity and spatial organization of the biofilm architecture from 5 to 20 h (Fig. 1A). During the initial stages of development, particularly within the first 2 h, we recorded a rapid increase in cell density within the lower layers of the biofilm, indicating concentrated microbial activity and growth at the surface. In Movie 1, these early basal-layer proliferations were confirmed, revealing rapid 3D expansion across the substratum followed by vertical thickening from 6 to 12 h. After 24 h biofilms reached in general a mature stadium and resembled in their overall appearance square sponges (Movie 1 and Fig. 1A).

Quantitative analysis (Fig. 1B) confirmed a significant shift in cell distribution over time, with increasing biofilm height and density observed between the 5-, 10-, 15-, and 20-h time points.

Thereby, biofilm volume increased by a factor of approximately 2.2 during the period from 8 to 24 h (Fig. 1C). The total biofilm volume exhibited a dynamic growth pattern (Fig. 1C): it initially increased until the 2-h mark, decreased slightly until 8 h, and subsequently rose again to reach a maximum of approximately 12,000 μm^3^ by 24 h. LLSM revealed that these fluctuations corresponded to cycles of microcolony expansion, contraction, and reorganization, underscoring the highly dynamic character of K279a biofilm architecture (Movie 1).

We established a negative correlation between cell density and biofilm height over time (Fig. 1D). Notably, cell density was consistently higher in the basal layers of the biofilm, although overall density was greater during the early stages of biofilm development. This relationship supported the conclusion that increased cell density contributed directly to the enhanced architectural complexity of the biofilm. These observations underscored the robust and dynamic biofilm-forming capabilities of *S. maltophilia* K279a under the tested conditions (Fig. 1D).

**Fig. 2:**
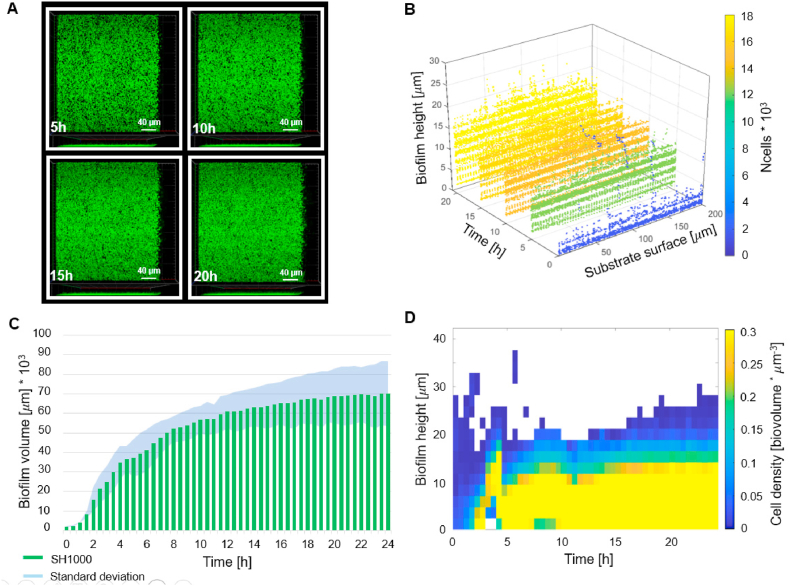
***Staphylococcus aureus* SH1000 develops structurally complex biofilms with dynamic spatial organization over 24 h.** Confocal laser scanning microscopy images illustrating the morphological changes of SH1000 biofilms at 5, 10, 15, and 20 h of growth. The images highlight the progressive maturation and structural complexity of the biofilms formed under static conditions in 10% LB at 37°C. **(B)** Quantification of cell distribution within a SH1000 biofilm at 5, 10, 15 and 20 h, indicating a shift in cell density and organization throughout the growth period. **(C)** The total biofilm volume of SH1000 measured from 0 to 24 h illustrates a dynamic growth pattern which demonstrates the robust characteristics of SH1000. **(D)** Correlation of cell density to biofilm height of SH1000 from 0 to 24 h, revealing a direct relationship between cell density and biofilm architecture. Microscopy was performed using a lattice light sheet microscope, enabling high-resolution, volumetric imaging of biofilm architecture with minimal phototoxicity. Quantification was performed using the software BiofilmQ. Together, these results provide insight into the dynamic growth characteristics and spatial organization of SH1000 biofilms under the experimental conditions tested.

We observed similar trends in biofilm development for *Staphylococcus aureus* SH1000. LLSM images revealed progressive biofilm maturation and increasing structural complexity over time, particularly between 5 and 20 h (Fig. 2A). Quantitative analysis demonstrated that cell density increased consistently throughout the biofilm structure, mirroring trends observed in *S. maltophilia* K279a (Fig. 2B). Movie 1 revealed dense, multilayered cluster formation as early as 4 h, with rapid vertical thickening and lateral spreading between 6 and 12 h. Unlike K279a, SH1000 biofilms showed sustained volumetric growth without collapse phases, indicating continuous consolidation.

We monitored biofilm growth for 24 h, and during the time period from 0 to 8 h, biofilm volume increased by a factor of approximately 6 (Fig. 2C). The total biofilm volume followed a characteristic growth curve, steadily rising and eventually reaching a maximum of approximately 70,000 μm^3^ by 24 h, substantially higher than the final volume recorded for *S. maltophilia*, indicating a greater biofilm-forming capacity in SH1000. LLSM confirmed this continuous increase, documenting persistent accretion of biomass and stabilization of the biofilm's basal layers after 12 h (Movie 1).

Throughout the 24-h period, *S. aureus* SH1000 maintained exceptionally high cell densities, particularly concentrated in the lower 10 μm layers of the biofilm (Fig. 2D). These dense basal layers exceeded those observed in *S. maltophilia* K279a underscoring species-specific differences. Notably, the layers of dense biofilm increased over time, indicating progressive thickening and accumulation in the basal regions.

These findings highlighted the robust and efficient biofilm formation of *S. aureus* SH1000 under the experimental conditions tested, offering insight into species-specific biofilm architecture and development dynamics.

**Fig. 3:**
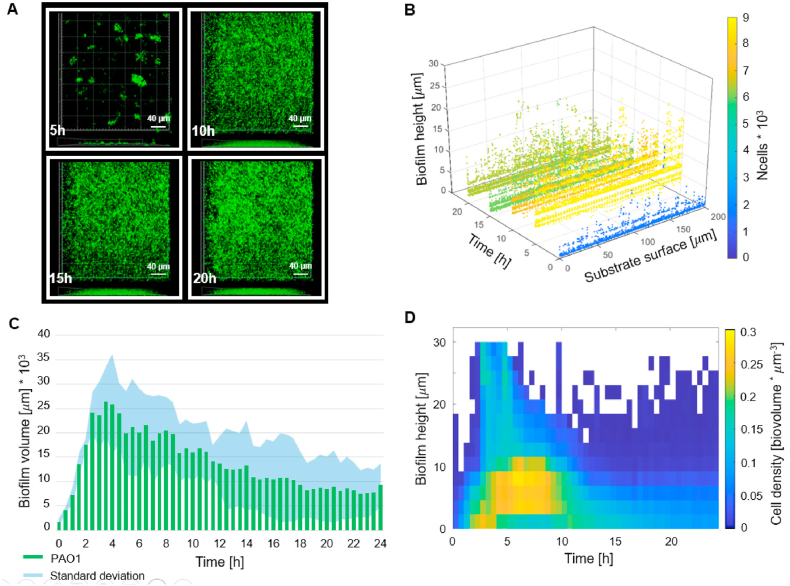
***Pseudomonas aeruginosa* PAO1 develops structurally complex biofilms with dynamic spatial organization over 24 h.** Confocal laser scanning microscopy images illustrating the morphological changes of PAO1 biofilms at 5, 10, 15, and 20 h of growth. The images highlight the progressive maturation and structural complexity of the biofilms formed under static conditions in 10% LB at 37°C. **(B)** Quantification of cell distribution within a PAO1 biofilm at 5, 10, 15 and 20 h, indicating a shift in cell density and organization throughout the growth period. **(C)** The total biofilm volume of PAO1 measured from 0 to 24 h illustrates a dynamic growth pattern which demonstrates the robust characteristics of PAO1. **(D)** Correlation of cell density to biofilm height of PAO1 from 0 to 24 h, revealing a direct relationship between cell density and biofilm architecture. Microscopy was performed using a lattice light sheet microscope, enabling high-resolution, volumetric imaging of biofilm architecture with minimal phototoxicity. Quantification was performed using the software BiofilmQ. Together, these results provide insight into the dynamic growth characteristics and spatial organization of PAO1 biofilms under the experimental conditions tested.

*Pseudomonas aeruginosa* PAO1 also exhibited substantial increases in biofilm volume and structural complexity over the 24-h incubation period. LLSM imaging revealed a clear biofilm maturation process from 5 to 20 h (Fig. 3A), yet the growth dynamics were distinct from those of *S. maltophilia* K279a and *S. aureus* SH1000. LLSM, however, revealed distinct growth kinetics: rapid volumetric expansion during the first 3-4 h, followed by progressive fragmentation of biofilm clusters. This suggested that PAO1 biofilms underwent early overgrowth followed by partial dispersal (Movie 1).

Quantitative assessments showed that biofilm volume increased rapidly during the early phase of development, reaching a peak at approximately 3.5 h. This was followed by a gradual decline in biofilm volume until the 24-h endpoint (Fig. 3C). We monitored biofilm growth for 24 h, and during the period from 0 to 3.5 h, biofilm volume increased by a factor of approximately 4.5 (Fig. 3C). Biofilm height data indicated a decrease from about 15 μm at 3.5 to approximately 10 μm by 24 h (Fig. 3B).

During the early stages of biofilm formation (2 to 9 h), cell density was highly concentrated within the lower 10 μm of the biofilm during early time points (Fig. 3D). A strong correlation between cell density and biofilm architecture was reaffirmed, emphasizing the interdependent nature of these parameters in PAO1 biofilm development.

Collectively, these results demonstrated that while both SH1000 and PAO1 form robust biofilms, the spatiotemporal dynamics of *S. maltophilia* K279a reveal a particularly dynamic and adaptive biofilm architecture.

Together, LLSM and biofilm quantification analyses illustrate that all three microbial species exhibit dynamic growth characteristics and complex spatial organization within their single-species biofilms under the experimental conditions tested. This comparative analysis underscores both shared and species-specific strategies of biofilm development. To facilitate comparison between the baseline single-species behaviors and the interaction patterns observed in dual-species biofilms, a condensed overview is provided in Table 2.

**Table 2:** Condensed comparison of single- and dual-species biofilm behavior.

System	Biomass dynamics	Spatial organization	Interaction outcome	Key mechanistic insight
**K279a**	Moderate biomass with cyclic remodeling	Dense basal growth, increasing height, sponge-like architecture	—	Highly dynamic, structurally adaptive biofilm former
**SH1000**	Strong continuous accumulation	Extremely dense basal layers, stable multilayer clusters	—	Efficient biomass producer with sustained consolidation
**PAO1**	Rapid early expansion followed by dispersal	Early basal dominance, later fragmentation and height reduction	—	Biofilm prone to overgrowth–dispersal transition
**K279a + SH1000**	K279a dominates; SH1000 biomass reduced	Vertical stratification (“sandwich”): K279a basal/apical, SH1000 intermediate	Coexistence via niche partitioning	K279a structures the biofilm and constrains partner expansion
**K279a + PAO1**	K279a dominant overall; PAO1 suppressed	Basal takeover by K279a; PAO1 confined to upper layers	Competitive segregation	K279a enforces spatial control and represses PAO1 QS/virulence programs

### 3.2 Challenging *S. maltophilia* biofilms with 100 μg Amp reveals a spatiotemporal expression of the β-lactamase gene

Previously, we demonstrated that the *bla2* gene is strictly induced by β-lactam antibiotics and that its expression occurs in a phenotypically heterogeneous manner within isogenic populations [24]. To extend these findings from planktonic cultures and colony morphotypes to structured communities, we constructed a bla2:sfGFP reporter fusion and employed the clinical isolate *S. maltophilia* SKK55 (Table 1). Carrying this fusion on the broad-host-range plasmid pBBR1-MCS enabled direct visualization of the spatiotemporal regulation of β-lactamase 2 under antibiotic stress in biofilms.

**Fig. 4:**
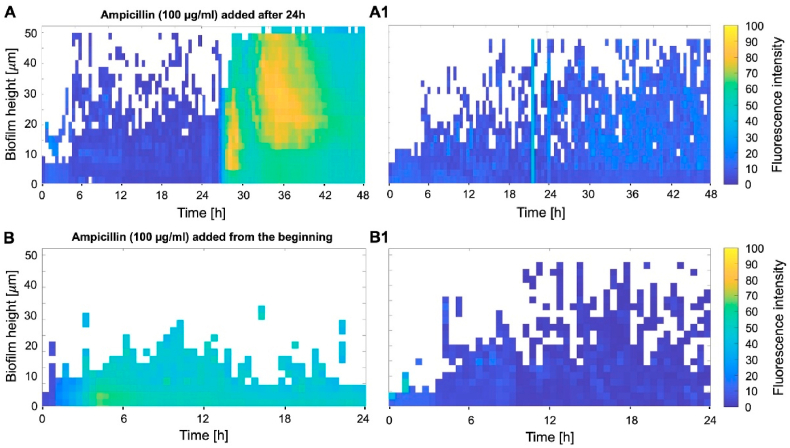
**Ampicillin alters biofilm formation and bla2 reporter expression in *Stenotrophomonas maltophilia* SKK55. (A)** Heatmap depicting biofilm height and fluorescence intensity of SKK55 harboring the reporter fusion pBBR1MCS:*bla2*::sfGFP after 24 h of growth. Following this period, Ampicillin was introduced, triggering the expression of the reporter fusion. (**A1)** Control heatmap showing biofilm height and fluorescence intensity of SKK55 with the same reporter fusion pBBR1MCS:*bla2*::sfGFP, but without the addition of Ampicillin. (**B**) Heatmap showing biofilm height and fluorescence intensity when Ampicillin (100 μg/ml) was present from the beginning of the experiment. In contrast to condition A, early exposure to the antibiotic led to reduced biofilm development and lower overall fluorescence intensity, indicating a distinct stress response and altered *bla2* expression dynamics. (**B1**) Control heatmap of SKK55 harboring the reporter fusion without any Ampicillin throughout the experiment, serving as a baseline for comparison. Microscopy was performed using a lattice light sheet microscope, enabling high-resolution, volumetric imaging of biofilm architecture with minimal phototoxicity. Quantification was performed using the software BiofilmQ. Overall, the heatmaps and confocal images illustrate the differential effects of Ampicillin on biofilm formation and fluorescence intensity in SKK55, highlighting the role of antibiotic-induced gene expression mediated by *bla2* in biofilm dynamics and the organism's resilience against antibiotic treatment.

When Ampicillin was added after 24 h of growth (Fig. 4A; Movie 1, Fig. S1), a rapid and pronounced increase in fluorescence was observed. This induction was restricted to the upper biofilm layers (30-40 μm in height) and became most pronounced between 30 and 40 h. Live imaging revealed that fluorescence spread laterally along the biofilm surface, indicating that cells directly exposed to the antibiotic gradient mounted the strongest *bla2* response. In contrast, the lower layers (<15 μm) showed little to no induction, suggesting that spatial heterogeneity in gene expression is tightly linked to antibiotic penetration depth. The control without Ampicillin (Fig. 4A1; Movie 2, no antibiotic) displayed only basal fluorescence, evenly distributed throughout the biofilm, confirming that induction is antibiotic dependent.

Supplementary data related to this article can be found online at https://doi.org/10.1016/j.bioflm.2026.100374

The following are the Supplementary data related to this article:

Multimedia component 9

By comparison, when Ampicillin was present from the beginning of the experiment (Fig. 4B; Movie 2, + Amp from start), biofilm growth was markedly impaired, with biomass (cell height) restricted to a level of ≤20 μm. Fluorescence remained weak and patchy, and no spatial stratification of *bla2* expression was detected. This suggests that early antibiotic exposure both suppresses biofilm development and alters the dynamics of resistance gene induction. The corresponding control (Fig. 4B1; Movie 2, -Amp) showed robust biofilm growth (∼40–50 μm) with only basal reporter activity.

Building on these insights into how antibiotic timing shapes single-species biofilm behavior, we next applied LLS microscopy with advanced cell-resolved analyses to investigate dual-species biofilms.

### 3.3 Dual-species biofilms: Differential growth dynamics and spatial distribution in 24 h biofilms of *S. maltophilia* with *S. aureus* or *P. aeruginosa*

Following the preceding section, in which the dynamics of single-species biofilm formation was analyzed for *S. maltophilia, P. aeruginosa* and *S. aureus* as well as the gene expression patterns of *S. maltophilia* in its single-species biofilm model, we then placed particular emphasis on the role of *S. maltophilia* in the dynamics and spatial organization of dual-species biofilm systems. Specifically, we investigated how *S. maltophilia* K279a interacts with either *S. aureus* SH1000 or *P. aeruginosa* PAO1.

We first analyzed biofilms formed by *S. maltophilia* K279a in coculture biofilms under static conditions with *S. aureus* SH1000. Confocal microscopy images (Fig. 1A) revealed dynamic spatial organization and structural evolution of the biofilm at 5, 10, 15, and 20 h under static conditions. These images indicated distinct spatial stratification and interspecies clustering. Quantitative biofilm volume analysis (Fig. 1B) revealed a biphasic growth pattern for K279a in coculture. Multispecies biofilm volume increased during the first 3 h, declined slightly until 8 h, and then resumed increasing steadily through 24 h. In contrast, SH1000 showed an initial increase until 2 h, after which its biofilm volume plateaued. Notably, SH1000's biofilm volume remained approximately 70–80% lower in volume than that of K279a throughout the experiment, underscoring the dominant growth of *S. maltophilia* and suggesting competitive inhibition of *S. aureus* expansion.

Cell density profiling (Fig. 1C) further elucidated spatial organization within the coculture. K279a exhibited its highest cell density in the basal biofilm layers (0–10 μm), followed by a less dense region between 15 and 30 μm, and then a renewed increase in density from 30 to 50 μm, particularly after 20 h. SH1000 showed peak density between 7 and 15 μm, suggesting vertical niche separation. These stratified patterns indicated spatial differentiation driven by both competition and cohabitation dynamics, with K279a maintaining structural control over the biofilm. In agreement with these microscopy-based observations, quantitative spatial distribution analysis using BiofilmQ (Fig. 6) demonstrated that K279a (red) consistently occupied both the basal and apical layers of the biofilm, whereas SH1000 (green) localized predominantly to the intermediate layers over time. This resulted in a characteristic multilayered architecture, where K279a enclosed SH1000 in distinct top and bottom niches. Such organization further supports vertical niche partitioning and highlights the dominance of *S. maltophilia* in controlling overall biofilm stratification.

Quantification of the 24h dual-species biofilm of K279a and PAO1 (Fig. 2B) showed a consistent increase in biofilm volume for both species across the 24-h period. Density profiling (Fig. 2C) showed that K279a maintained relatively uniform density throughout the entire 24-h period, with a localized spike in density at approximately 40 μm height between 12.5 and 15 h that suggested transient cluster formation. In contrast, PAO1 displayed very low biofilm density across all time points and spatial layers, reflecting its suppressed biofilm-forming ability in the presence of K279a. A key observation is that although PAO1 initially predominates in the basal layer during the early stages of biofilm formation, it is later displaced by K279a, which ultimately establishes dominance in this layer. This surface positioning appears to trigger adaptive responses in PAO1, such as the downregulation of type IV pili, which are crucial for surface motility and initial adhesion. The diminished expression of these pili suggests that PAO1 is adapting to a more sessile, stable phenotype in response to K279a′s dominant spatial position, which possibly provides structural or protective advantages. This was further corroborated by vertical abundance profiles derived from BiofilmQ (Fig. 8), which revealed that K279a (red) became increasingly confined to the lower layers of the biofilm over time, while PAO1 (green) dominated the upper and middle regions. Unlike the structured vertical zonation observed with *S. aureus*, the K279a-PAO1 interaction exhibited more direct vertical exclusion, with PAO1 gradually outcompeting K279a in the upper strata despite its overall lower biomass contribution. This indicates spatial segregation dynamics driven by competitive or exclusion-based interactions.

Together, these findings highlighted distinct interaction dynamics in dual-species biofilms. In both coculture systems, *S. maltophilia* K279a emerged as the dominant biofilm former: with *S. aureus*, it established layered niche separation in a stratified pattern, while with *P. aeruginosa*, it showed strong basal colonization but was spatially excluded from upper biofilm regions. These results underscore the central role of *S. maltophilia* in shaping biofilm architecture and function in multispecies communities.

### 3.4 *S. maltophilia* K279a enforces spatial dominance and quorum sensing suppression in *P. aeruginosa PAO1* during dual-species biofilm growth

The observed differences in growth dynamics and spatial organization highlight the dominant role of *S. maltophilia* K279a in both dual-species settings, with species-specific consequences for its partners. Whereas co-culture with *S. aureus* SH1000 resulted in vertical niche partitioning that supported biofilm coexistence, interaction with *P. aeruginosa* PAO1 was marked by pronounced spatial dominance of K279a and suppression of PAO1 biofilm development. These findings raise the question of whether K279a not only dictates structural organization but also modulates the molecular regulatory responses of PAO1.

To validate and extend these observations, we re-analyzed transcriptomic datasets from our previous study in which *Stenotrophomonas maltophilia* K279a and *Pseudomonas aeruginosa* PAO1 were co-cultivated under identical experimental conditions in dual- and multispecies biofilms for 72 h. These data provided a valuable framework to assess how interspecies interactions within structured biofilms influence global gene expression patterns and metabolic adaptation.

In the data sets, we observed significant downregulation (≤−2 log2 fold change) of key QS-affected genes in PAO1, type VI pili (−2.33), outer membrane porin F (OprF) (−3.60), outer membrane lipoprotein (OprI) (−4.48), and cyclic di-GMP phosphodiesterase (−2.45) (Fig. S6). Notably, such downregulation was absent in single-species PAO1 biofilms, underscoring the unique influence exerted by the presence of K279a. Transcriptomic profiling after 72 h of static co-culture (Figs. S2 and S3) further implied that K279a suppresses QS regulators, including *lasA*, *lasB*, *lasI*, *lasR, rhlR*, and *vqsM,* underscoring the repression of virulence pathways and central QS hierarchies in dual-species biofilms. The *lasA* gene (−2.48) encodes a secreted protease that contributes to tissue degradation and immune evasion, whereas lasB (−5.95) encodes an elastase that plays a central role in host protein breakdown and nutrient acquisition. The *lasI* gene (−4.07) encodes the autoinducer synthase responsible for generating N-3-oxododecanoyl-homoserine lactone (3-oxo-C12-HSL), the signal molecule for the Las quorum-sensing system, while its cognate receptor LasR (−2.37) is a transcriptional regulator that activates multiple downstream virulence genes in response to this signal. Likewise, rhlR (−2.54) encodes the receptor of the second AHL-based QS circuit (Rhl system), which modulates factors such as rhamnolipid biosurfactants and additional virulence determinants. Finally, vqsM (−4.05) functions as a global master regulator that integrates quorum sensing with other regulatory pathways, reinforcing the broad suppression of QS hierarchies observed in dual-species biofilms (Gomes-Fernandes et al., 2022; Dehbashi et al., 2020.)

Within the Las system, *LasI* (AHL synthase), LasB elastase, LasA protease, and the alginate regulator AlgZ/FimS were all significantly downregulated (−5.9 to −2.4). Likewise, both PAO1 encoded lectins PA-I (LecA) and PA-IIL (LecB) showed significantly reduced transcription (−3.7). In the Rhl system, repression was observed for *rhlI* (AHL synthase), the type IV fimbrial protein FimU, alginate regulators MucA/AlgU, phenazine biosynthesis gene *phzC*, and a cyclic di-GMP phosphodiesterase (biofilm dispersal regulator). The Pqs system was also strongly affected, with -downregulation of *pqsB* (PQS biosynthesis enzyme) (−2.2), *phz*C (shared with the Rhl system), and PAAR4 (a T6SS spike protein). Finally, additional regulators such as an acyl-homoserine-lactone synthase of unknown function (−3.1) and the HTH-type transcriptional regulator VqsM (−4.1) were strongly repressed. Collectively, these findings indicate that K279a exerts widespread inhibitory effects on QS hierarchies, virulence determinants, and biofilm dispersal functions of PAO1 (Fig. S3).

Intriguingly, PAO1, pellicle biosynthesis genes PA3060-PA3063 were strongly upregulated (Log 2 fold change −2.5 to −2.1) in the dual-species biofilms together with genes involved in aromatic compound metabolism PA2512-PA2517 (Log 2 fold change −3.0 to −2.3).

Our previous transcriptome analysis (RNA-Seq) of dual-species biofilms comprising *S. maltophilia* K279a and either *P. aeruginosa* PAO1 or *S. aureus* SH1000 demonstrated that both species respond to the presence of the other by modulating their gene expression profiles. This transcriptional response of *S. maltophilia* was most pronounced in the *S. maltophilia* - *P. aeruginosa* dual biofilms, where 3.3% of genes in *S. maltophilia* were differentially regulated. Among the differentially regulated genes was the hemophore-encoding gene *smlt2713*, which has been previously characterized as playing a pivotal role in iron acquisition within *S. maltophilia* biofilms. Furthermore, the cytochrome *c* oxidase *smlt4401* was significantly upregulated in dual-species biofilms with *P. aeruginosa* PAO1, suggesting a functional adaptation in respiration in response to the dual species environment ([21],2023).

Consequently, this study utilizes reporter fusion constructs of these 2 genes to investigate the spatially and temporally resolved gene expression of *S. maltophilia*, focusing primarily on its interaction with *P. aeruginosa* in dual-species biofilms.

The response of *S. maltophilia* K279a in dual-species biofilms with *S. aureus* SH1000 was negligible, with only 0.5% of genes showing differential expression. The dynamics of *S. maltophilia* K279a gene expression in dual species biofilms with *S. aureus* SH1000 was therefore not further investigated in this study.

### 3.5 Interspecies interaction with *P. aeruginosa* enhances spatiotemporal expression of the cytochrome *c* oxidase gene smlt4401 in *S. maltophilia* K279a biofilms

We next investigated how interspecies interactions influence the gene regulatory networks of K279a itself. Given the pronounced impact of K279a on the spatial dynamics and transcriptional behavior of its partners, we hypothesized that reciprocal modulation may occur, with competing species shaping K279a′s metabolic and respiratory programs.

To identify genes potentially regulated by interspecies signals, we first performed an initial screening using a collection of 10 *S. maltophilia* reporter fusion constructs (Table 1) and incubated them with biofilm-derived supernatants from *P. aeruginosa* PAO1, K279a co-cultivated with PAO1 and PAO1 cells as outlined in Figs. S4 and S5. These reporter constructs had been generated as described in the Materials and Methods section.

Fluorescence signals were recorded, quantified, and normalized to cell density over 24, 48, and 72 h to assess changes in gene expression under these conditions. This first screening revealed variable responses across constructs, with some promoters, such as smlt2713, showing substantial upregulation upon exposure to *P. aeruginosa* supernatant. In contrast, smlt4401, encoding a cytochrome *c* oxidase subunit, displayed limited induction in response to supernatant exposure, suggesting that its regulation may require physical interaction or biofilm context. Despite the modest response of smlt4401 to cell-free *P. aeruginosa* supernatants, we selected this gene for further investigation using live-cell imaging, based on its predicted role in respiration and energy metabolism. We hypothesized that upregulation of smlt4401 might be contact-dependent and occur specifically within the biofilm microenvironment.

To test this, we tracked the spatiotemporal expression of *smlt4401* using a fluorescent reporter fusion construct (pBBR1MCS:*smlt4401*::mScarlet) in mono- and dual-species biofilms with *P. aeruginosa* PAO1.

For this, K279a and PAO1 were co-inoculated from the beginning of the experiment and at equal cell numbers (Fig. 9, Fig. S6). A progressive increase in fluorescence intensity, particularly from 24 h onward, was observed, coinciding with biofilm thickening and spatial stratification. The confocal images (A1), taken at 29 h, reveal strong mScarlet fluorescence throughout the biofilm, indicating active *smlt4401* expression in response to co-culture with PAO1.

In contrast, in control mono-species biofilm of K279a harboring the same reporter construct but grown without PAO1 (Fig. 9B) only weak fluorescence signals of *smlt4401 were observed.* The associated heatmap and confocal imaging (B1) indicated less structured biofilm architecture, reflecting baseline expression levels of *smlt4401* in the absence of interspecies interaction.

These results imply that the presence of *P. aeruginosa* PAO1 from the onset of biofilm formation significantly enhances the expression of the cytochrome *c* oxidase gene *smlt4401* in K279a. This further suggests that interspecies interactions within the biofilm microenvironment modulate respiratory gene regulation, potentially as an adaptive response to competition or altered redox conditions - regulatory features that may not be captured in cell-free supernatant-based assays.

### 3.6 *P. aeruginosa* enhances spatiotemporal expression of the hemophore gene *smlt2713* in *S. maltophilia* sm454 biofilms

Building on these observations of respiratory gene regulation, we next sought to determine whether interspecies interactions also influence other adaptive functions beyond energy metabolism. Since nutrient acquisition, particularly iron and heme scavenging, represents a critical determinant of survival within polymicrobial biofilms, we turned our attention to the expression dynamics of *smlt2713*, which encodes a hemophore HphA-like protein. This gene was selected for further analysis based on both its strong upregulation in dual-species transcriptomic data sets (K279a + PAO1, Alio et al., 2023) and its marked induction in the initial supernatant-based screening (Fig. S4).

Live/dead staining of biofilms followed by CLSM imaging showed clearly that the deletion of the *smlt2713* gene in *S. maltophilia* sm454 resulted in impaired Biofilm formation under Iron limitation (Fig. S7). A 45% decrease in biofilm volume was observed for the mutant strain as compared to wild type and this decrease was associated with the live cell fraction in the biofilms.

The *smlt2713* reporter strain showed consistently elevated expression in response to *P. aeruginosa* biofilm supernatant at multiple time points, suggesting it is highly responsive to secreted interspecies signals related to iron stress or heme competition.

To evaluate the influence of *P. aeruginosa* PAO1 on *smlt2713* expression during biofilm development, we employed a new fluorescent reporter fusion construct (pBBR1MCS:smlt2713:mScarlet) in *S. maltophilia* strain sm454. Biofilms were grown under static conditions in 10% LB medium at 37°C for 72 h, either in the presence or absence of PAO1-sfGFP.

Co-cultivation with PAO1 resulted in a strongly and significantly increase in biofilm-associated fluorescence over time, particularly between 25 and 40 h, reaching a peak intensity within the mid-layer of the biofilm (∼20-35 μm in height) (Fig. 10A, Figure S8 A). This fluorescence pattern suggested a temporal and spatial regulation of *smlt2713*-driven expression in response to the presence of PAO1. The corresponding confocal laser scanning microscopy images (A1), taken after 24 h, revealed a dense biofilm structure with prominent mScarlet fluorescence, indicative of active reporter gene expression.

In contrast, the controls lacking PAO1 (Fig. 10B, Figure S8 B) showed substantially lower and more sporadic reporter fluorescence throughout the biofilm over the same time course. The heatmap indicated only minor fluorescence signals, primarily early during biofilm development (<20 h), with no sustained signal over time. The corresponding confocal image (B1) revealed a similar overall biofilm architecture but with markedly reduced fluorescence, supporting the heatmap data and suggesting that the reporter gene is minimally expressed in the absence of PAO1.

Together, these results strongly imply that the presence of PAO1 significantly enhances expression of the *smlt2713* gene in sm454 biofilms. Given that *smlt2713* encodes a hemophore HphA-like protein that is clearly involved in iron acquistion, these findings implicate interspecies interactions in modulating heme-related gene expression and iron acquisition processes during biofilm development. Unlike *smlt4401*, which appears to require direct interspecies contact or structured biofilm formation for its upregulation, *smlt2713* responds robustly to soluble factors.

## 4 Discussion

### 4.1 Spatial organization and biofilm architecture

This study uncovers novel details on how *S. maltophilia* K279a builds up single species biofilms and how it tends to dominate the basal layers of dual-species biofilms when co-cultured with the two clinically relevant pathogens, *P. aeruginosa* and *S. aureus*. Using high resolution live cell imaging we monitored over time the classical developmental stages for *S. maltophilia* K279a, *P. aeruginosa* PAO1*,* and *S. aureus* SH1000 in singles and dual species biofilms.

We further provide strong evidence that *S. maltophilia* K279a develops heterogeneous 3D microcolony biofilms within 24 h, a feature not well characterized before, but one that highlights its structural diversification and aligns it with other opportunistic pathogens [[33], [34], [35], [36]].

**Fig. 5:**
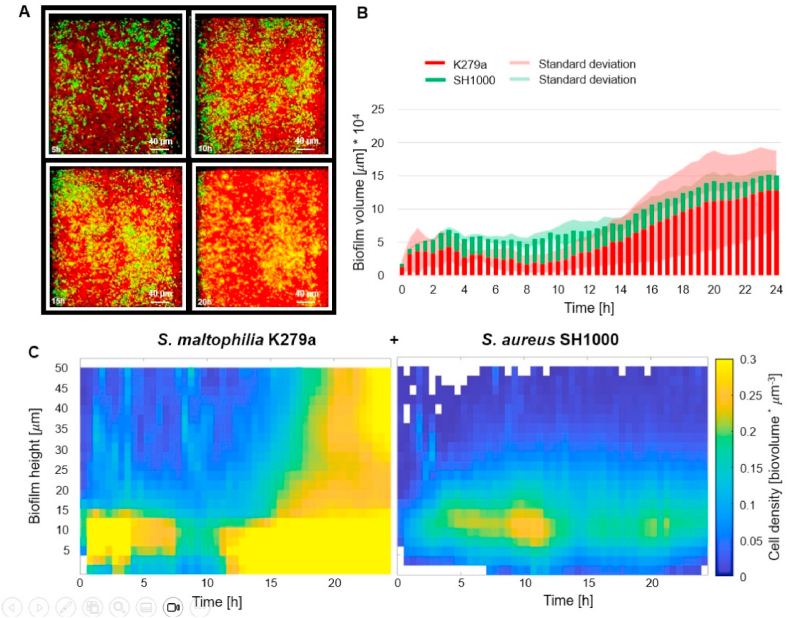
***Stenotrophomonas maltophilia* K279a and *Staphylococcus aureus* SH1000 form dual-species biofilms with dynamic growth and spatial organization. (A)** Microscopy images illustrating the spatial distribution and growth morphology of the dual species biofilm *S. maltophilia* K279a and *S. aureus* SH1000 at 5, 10, 15, and 20 h at 37° in 10% LB under static conditions. **(B)** Quantification of biofilm volume (μm^3^) of the dual species biofilm K279a and SH1000 over a 24-h period. The graph demonstrates the dynamic growth patterns of the dual species biofilm, highlighting a significant increase in volume as a function of time. **(C)** Density profiling of the dual species biofilm K279a and SH1000 over time. The left panel shows the density measurements of K279a throughout the co-culture period (0 to 24 h), while the right panel presents the corresponding density of SH1000. Microscopy was performed using a lattice light sheet microscope, enabling high-resolution, volumetric imaging of biofilm architecture with minimal phototoxicity. Quantification was performed using the software BiofilmQ. All panels provide insights into the competitive and synergistic interactions during biofilm development as well as niche formation.

**Fig. 6:**
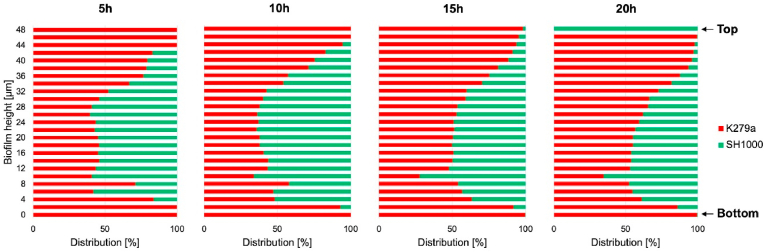
***Stenotrophomonas maltophilia* K279a and *Staphylococcus aureus* SH1000 establish stratified spatial organization in dual-species biofilms over time.** Quantification of dual-species biofilm architecture using BiofilmQ. Bar plots show the vertical distribution of each species within the biofilm at 5 h, 10 h, 15 h, and 20 h. *S. maltophilia* K279a is shown in red and *S. aureus* SH1000 in green. The y-axis represents biofilm height (0-48 μm), from bottom to top, and the x-axis indicates the relative abundance of each species at each height interval. Microscopy was performed using a lattice light sheet microscope, enabling high-resolution, volumetric imaging of biofilm architecture with minimal phototoxicity. Quantification was performed using the software BiofilmQ. Over time, a characteristic vertical zonation emerges: *S. maltophilia* K279a predominates in the bottom and top layers of the biofilm, while *S. aureus* SH1000 occupies the intermediate layers. This stratified organization suggests spatial structuring and potential niche partitioning between the two species during biofilm development. (For interpretation of the references to colour in this figure legend, the reader is referred to the Web version of this article.)

**Fig. 7:**
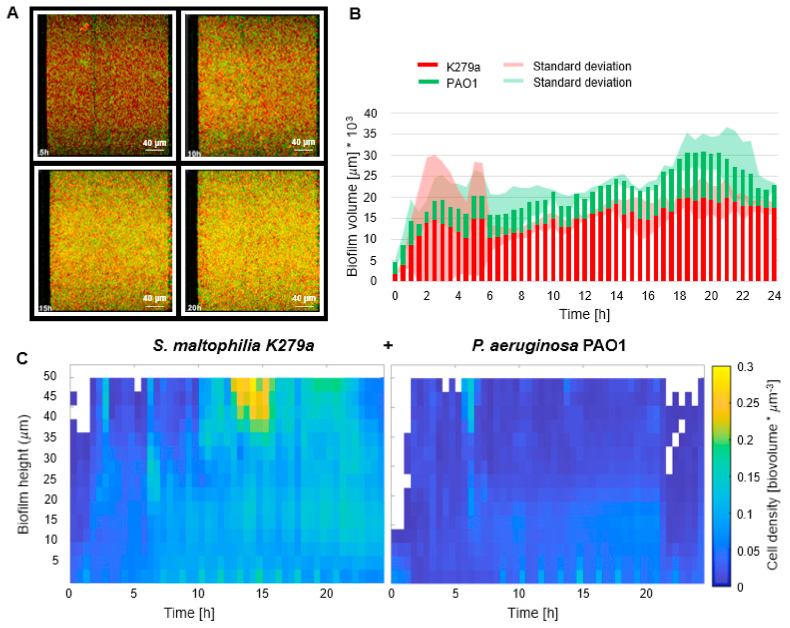
***Stenotrophomonas maltophilia* K279a and *Pseudomonas aeruginosa* PAO1 form dual-species biofilms with dynamic growth and spatial organization. (A)** Microscopy images illustrating the spatial distribution and growth morphology of the dual species biofilm *S. maltophilia* K279a and *P. aeruginosa* PAO1 at 5, 10, 15, and 20 h at 37° in 10% LB under static conditions. **(B)** Quantification of biofilm volume (μm^3^) of the dual species biofilm K279a and PAO1 over a 24-h period. The graph demonstrates the dynamic growth patterns of the dual species biofilm, highlighting a significant increase in volume as a function of time. **(C)** Density profiling of the dual species biofilm K279a and PAO1 over time. The left panel shows the density measurements of K279a throughout the co-culture period (0 to 24 h), while the right panel presents the corresponding density of PAO1. Microscopy was performed using a lattice light sheet microscope, enabling high-resolution, volumetric imaging of biofilm architecture with minimal phototoxicity. Quantification was performed using the software BiofilmQ. All panels provide insights into the competitive and synergistic interactions during biofilm development as well as niche formation.

**Fig. 8:**
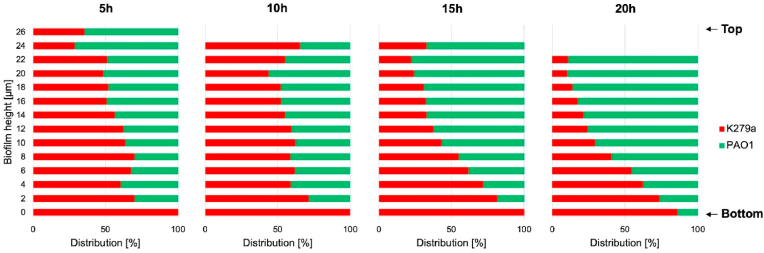
***Stenotrophomonas maltophilia* K279a and *Pseudomonas aeruginosa* PAO1 display stratified spatial organization in dual-species biofilms over time.** Quantification of dual-species biofilm structure using BiofilmQ. Bar plots display the vertical distribution of *S. maltophilia* K279a (red) and *P. aeruginosa* PAO1 (green) at 5 h, 10 h, 15 h, and 20 h. The y-axis represents biofilm height (0–26 μm), from bottom to top, and the x-axis indicates the relative abundance of each species at each height layer. Microscopy was performed using a lattice light sheet microscope, enabling high-resolution, volumetric imaging of biofilm architecture with minimal phototoxicity. Quantification was performed using the software BiofilmQ. Over time, *P. aeruginosa* PAO1 increasingly dominates the upper and middle regions of the biofilm, while *S. maltophilia* K279a becomes confined primarily to the lower layers. This vertical stratification suggests competitive or spatial exclusion dynamics between the two species during biofilm maturation. (For interpretation of the references to colour in this figure legend, the reader is referred to the Web version of this article.)

In our 24 h dual-species biofilms, *S. maltophilia* K279a consistently emerged as the dominant colonizer, clearly outperforming its competitors *P. aeruginosa* PAO1 and *S. aureus* SH1000 (Fig. 5, Fig. 6, Fig. 7, Fig. 8 & 11, MOVIE 4). This dominance underscores its pathogenicity and competitive abilities [37,38]. In combination with *S. aureus*, it created a multilayered stratification in which *S. maltophilia* occupied both basal and apical layers, confining *S. aureus* to an intermediate zone.

**Fig. 9:**
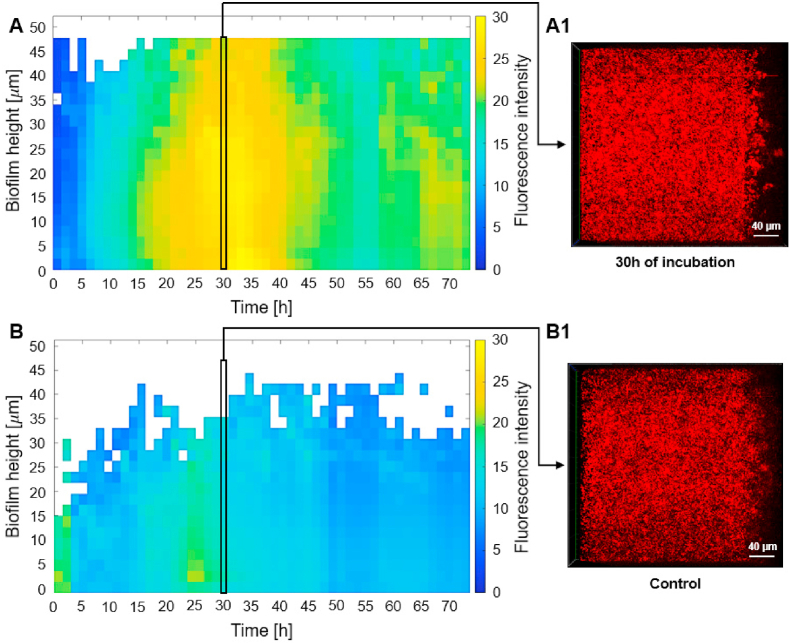
***Pseudomonas aeruginosa* PAO1 modulates biofilm formation and smlt4401 (cyoA) reporter expression in *Stenotrophomonas maltophilia* K279a. (A)** Heatmap depicting biofilm height and fluorescence intensity of K279a harboring the reporter fusion pBBR1MCS:*smlt4401*::mScarlet which was cocultivated with PAO1 sfGFP between 0 and 72 h of growth. (**A1)** Confocal laser scanning image illustrating the biofilm structure under static conditions at 37°C in 10% LB medium, taken after 24 h of growth, demonstrating enhanced fluorescence indicative of reporter expression. **(B)** Control heatmap showing biofilm height and fluorescence intensity of K279a with the same reporter fusion pBBR1MCS:*smlt4401*::mScarlet, but without the addition of PAO1 sfGFP. (**B1)** Confocal laser scanning image of the biofilm grown under static conditions at 37°C in 10% LB medium for 24 h, revealing the biofilm's structural characteristics in the absence of PAO1 sfGFP. Microscopy was performed using a lattice light sheet microscope, enabling high-resolution, volumetric imaging of biofilm architecture with minimal phototoxicity. Quantification was performed using the software BiofilmQ. Overall, the heatmaps and confocal images illustrate the differential effects of PAO1 on biofilm formation and fluorescence intensity in K279a, highlighting the role of PAO1 induced gene expression mediated by *smlt4401*in biofilm respiration.

**Fig. 10:**
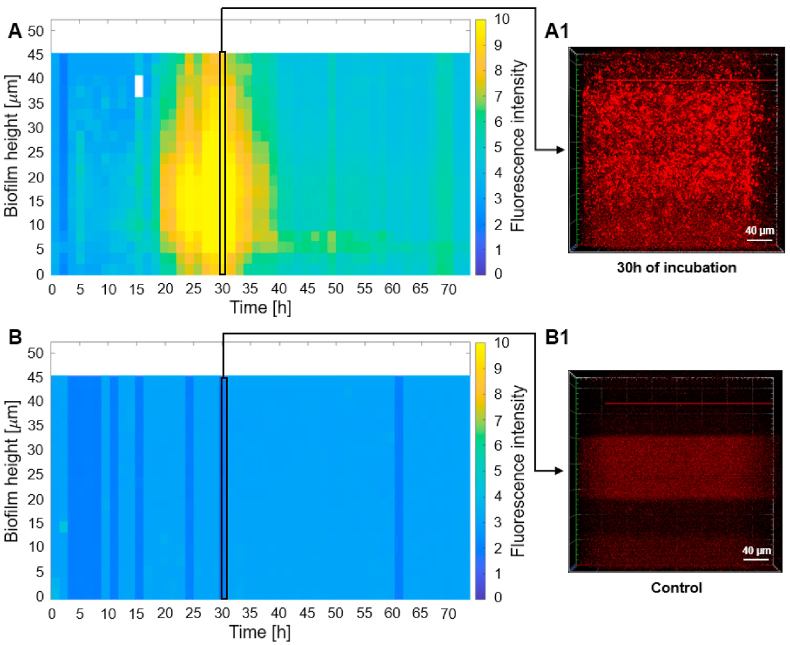
***Pseudomonas aeruginosa* PAO1 modulates biofilm formation and smlt2713 reporter expression in *Stenotrophomonas maltophilia* sm454. (A)** Heatmap depicting biofilm height and fluorescence intensity of sm454 harboring the reporter fusion pBBR1MCS:*smlt2713*::mScarlet which was cocultivated with PAO1 sfGFP between 0 and 72 h of growth. (**A1)** Confocal laser scanning image illustrating the biofilm structure under static conditions at 37°C in 10% LB medium, taken after 24 h of growth, demonstrating enhanced fluorescence indicative of reporter expression. **(B)** Control heatmap showing biofilm height and fluorescence intensity of sm454 with the same reporter fusion pBBR1MCS:*smlt2713*::mScarlet, but without the addition of PAO1 sfGFP. (**B1)** Confocal laser scanning image of the biofilm grown under static conditions at 37°C in 10% LB medium for 24 h, revealing the biofilm's structural characteristics in the absence of PAO1 sfGFP. Microscopy was performed using a lattice light sheet microscope, enabling high-resolution, volumetric imaging of biofilm architecture with minimal phototoxicity. Quantification was performed using the software BiofilmQ. Overall, the heatmaps and confocal images illustrate the differential effects of PAO1 on biofilm formation and fluorescence intensity in sm454, highlighting the role of PAO1 induced gene expression mediated by *smlt2713*.

(Fig. 11) With *P. aeruginosa*, *S. maltophilia* maintained basal dominance, while PAO1 gradually established itself in the upper layers (Fig. 5, Fig. 6, Fig. 7, Fig. 8 & 11, MOVIE 4). To highlight these spatial organizations, Fig. 11 was introduced. These findings echo previous reports on vertical niche partitioning as a coexistence strategy in multispecies biofilms [39], but extend them by showing that *S. maltophilia* enforces basal dominance and thereby dictates spatial organization.

**Fig. 11:**
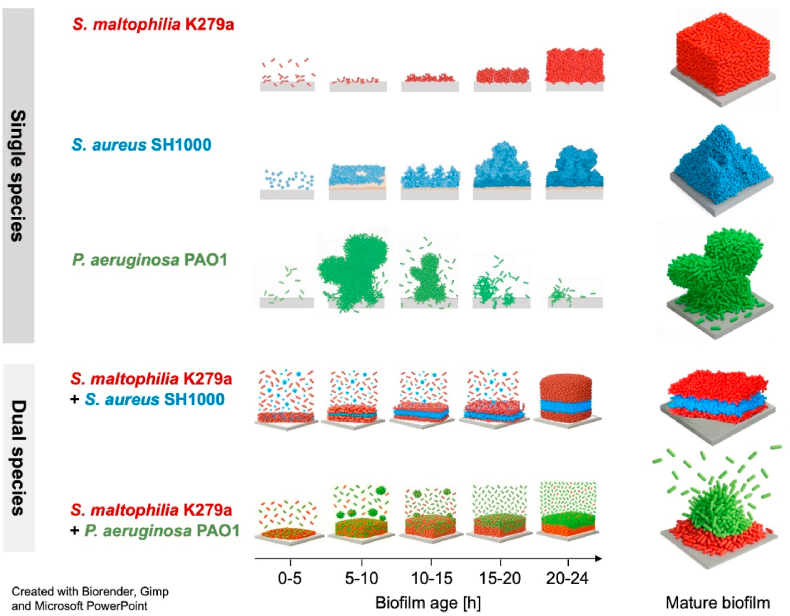
**Schematic overview of single- and dual-species biofilm development over 24h at 37°C in 10% LB medium under static conditions.** Biofilm formation is shown for *S. maltophilia* K279a (red), *S. aureus* SH1000 (blue), and *P. aeruginosa* PAO1 (green). In single-species cultures, K279a develops dense, vertically stacked structures, SH1000 forms compact aggregates, and PAO1 generates irregular biofilms prone to dispersal. In dual-species cultures, K279a and SH1000 assemble into a stratified layering, with K279a occupying basal and upper layers and SH1000 localized in the intermediate zone. In contrast, K279a-PAO1 biofilms, K279a dominates the basal layer during initial development, while PAO1 colonizes the upper regions and undergoes dispersal at later stages. More detailed analyses of single-species biofilms are provided in Fig. 1, Fig. 2, Fig. 3, and of dual-species biofilms in Fig. 5, Fig. 6, Fig. 7, Fig. 8. (For interpretation of the references to colour in this figure legend, the reader is referred to the Web version of this article.)

Supplementary data related to this article can be found online at https://doi.org/10.1016/j.bioflm.2026.100374

The following are the Supplementary data related to this article:

Multimedia component 11

### 4.2 Spatiotemporal gene regulation and antibiotic response

One of the major findings of this study is the characterization of the spatiotemporal gene expression profile of *S. maltophilia* within biofilms. Exposure of *S. maltophilia* biofilms to Ampicillin elicited a clear response, marked by the upregulation of the β-lactamase gene predominantly in the upper biofilm layers reflecting the role of diffusion gradients in antibiotic tolerance [40]. Although ampicillin is not considered a first-line therapeutic agent for *Stenotrophomonas maltophilia* infections, the bla2 reporter fusion construct employed in this study serves as a model system to investigate the spatial and temporal expression dynamics of antibiotic resistance genes within biofilms under antibiotic exposure.

Similar observations have been reported for *P. aeruginosa* biofilms, in which β-lactam antibiotics such as imipenem and ceftazidime induced β-lactamase expression that was largely confined to the periphery of microcolonies [41].

Our experiments showed that when Ampicillin was present from the start, biofilm development was suppressed, biofilms were patchy and weak. These findings highlight how *S. maltophilia* employs localized and temporally dynamic gene regulation to withstand antimicrobial stress.

### 4.3 Iron acquisition and metabolic competition

We also detected a time-dependent increase in the expression of the iron-scavenging protein smlt2713 in dual-species biofilms with *P. aeruginosa*. Comparable patterns of spatiotemporal gene regulation have been described for biofilm communities of *Vibrio cholerae* and *Bacillus subtilis* [42,43]

Taken together, this heterogeneity in gene expression across biofilm structures suggests division of labor and cooperative interactions among spatially distinct cell subpopulations, ultimately enhancing overall biofilm fitness.

### 4.4 Quorum sensing interference and competitor suppression

Previous research has emphasized antagonistic interactions between *P. aeruginosa* and *S. aureus* and the persistence of *S. maltophilia* in polymicrobial niches, especially in cystic fibrosis lungs [44,45]. Our findings add to this by showing that *S. maltophilia* is not a passive colonizer but actively reshapes both the biofilm architecture and transcriptomic profiles of its neighbors, consistent with earlier reports of competitive exclusion in mixed microbial communities [46,47] (Alio & Moll, 2023).

A striking effect we observed was the attenuation of *P. aeruginosa* quorum sensing (QS). In co-culture, *S. maltophilia* downregulated *las* and *rhl* QS hierarchies, translating into reduced virulence and motility. This mirrors observations that other microbial competitors can interfere with *P. aeruginosa* QS [48]. Quorum sensing also plays a major role in the attachment of bacteria to surfaces, cell-surface or cell-cell adhesions. This has been reported for a variety of other species such as *V.cholera, E. coli, L. monocytogenes, Burkholderia cenocepacia* etc [49].

Suppression of motility and attachment associated genes in PAO1 after 72 h of co-culture was also observed, suggesting that *P. aeruginosa* attachment to the surface is impaired in the presence of *S. maltophilia* and therefore undergoes an adaptive shift toward a less surface-associated phenotype under this competitive stress. This allows *S. maltophilia* to dominate and colonize the bottom layer of the biofilms.

Iron acquisition emerged as another central factor driving interspecies interactions. Strong upregulation of the hemophore gene *smlt2713* (log 2 fold change 4.9) in the presence of *P. aeruginosa* mirrors prior reports that iron competition governs spatial segregation in multispecies biofilms [[50], [51], [52]].

The reduced biofilm volume observed in the deletion mutant *S. maltophilia* 454 Δ smlt2713 under iron-limited conditions clearly indicates that iron plays a critical role in biofilm formation. Consistent with this observation, our previous study [21] demonstrated a strong upregulation of smlt2713 expression in biofilm-associated cells compared with planktonic cultures (log2 fold change >10).

Previous reports have also shown that iron-rich media significantly enhance both biofilm formation and growth rates in *P. aeruginosa*, suggesting that environmental iron availability promotes biofilm development [53].

Taken together, these findings suggest that, within the dual-species biofilm formed by *S. maltophilia* 454 and *P. aeruginosa* PAO1, both organisms compete for the available iron. This competition may, in turn, induce the upregulation of *smlt2713* in *S. maltophilia* which we observed in our reporter strain.

Induction of respiratory genes such as *smlt4401* further suggests that redox stress and metabolic competition shape *S. maltophilia*'s adaptive responses. Comparable mechanisms have been described in *Acinetobacter baumannii*, where iron limitation drives hemophore expression in mixed species biofilms [54].

### 4.5 Intracellular signaling and stabilization of spatial niches

Cyclic di-GMP signaling also revealed species-specific adaptations. In PAO1, repression of phosphodiesterase genes indicated sustained c-di-GMP levels, reinforcing biofilm behaviors but confining the bacterium spatially to upper regions. Conversely, *S. maltophilia* upregulated diguanylate cyclase in late-stage co-culture, enhancing its own c-di-GMP levels and stabilizing basal colonization. Such asymmetric modulation of c-di-GMP signaling echoes broader findings that interspecies interactions can drive species-specific intracellular signaling shifts in mixed biofilms, reinforcing spatial segregation as a strategy for coexistence [51].

### 4.6 Integrated strategy of ecological dominance and clinical implications

Together, our results suggest that *S. maltophilia*'s ecological success in polymicrobial biofilms relies on a multifaceted strategy: This includes at least suppression of *P. aeruginosa* QS, motility, and membrane integrity, preferential colonization of basal biofilm layers, induction of cyclic di-GMP, respiratory, iron-scavenging functions and spatiotemporally restricted antibiotic resistance gene expression. These mechanisms confer both structural and metabolic dominance, explaining its persistence in chronic infections. By monopolizing heme and iron, suppressing competitors' virulence, and reinforcing its biofilm core, *S. maltophilia* can thrive in environments of high competition. Recent evidence that it contributes extracellular matrix components to adhesion and displacement of rivals [10,55] further supports our observations.

Overall, *S. maltophilia* secures ecological dominance through integrated structural, metabolic, and resistance strategies. Its attenuation of *S. aureus* growth and strong suppression of *P. aeruginosa* virulence, monopolization of heme and iron, and fine-tuned antibiotic responses highlight its clinical relevance as a resilient pathogen.

## 5 Conclusions

This study shows that *S. maltophilia* exerts dominant, multifaceted control in 24 h dual-species biofilms with *P. aeruginosa* and *S. aureus*. *S. maltophilia* establishes itself as the primary colonizer, reshaping spatial structure, gene regulation, and survival of competitors. With *S. aureus*, it forms stratified architectures, while with *P. aeruginosa* it enforces niche displacement by suppressing motility-, adhesion-, and membrane-related genes.

In turn, *S. maltophilia* adapts by upregulating respiratory and iron-acquisition genes, enhancing diguanylate cyclase activity, and promoting matrix production. Antibiotic challenges reveal layer-specific β-lactamase induction, reflecting localized and regulated resistance.

These findings provide a basis for future studies aimed at developing strategies to target complex multispecies biofilms in clinical settings.

## AI declaration statement

The authors declare that generative AI tools were used in the preparation of this manuscript solely to improve the clarity, readability, and scientific language of the text. Specifically, ChatGPT (OpenAI) and Gemini (Google) were employed to assist with grammar refinement, sentence structure, and improving overall readability. All content, interpretations, and conclusions remain the sole responsibility of the authors.

The authors confirm that the AI tool was used under human oversight and control, and that every section of the manuscript was carefully reviewed, edited, and validated to ensure accuracy, originality, and alignment with the authors’ own analysis and insights. No AI-generated content was accepted without thorough verification, and the tool was not used to generate or fabricate data, references, analyses, or scientific conclusions.

The authors affirm that the use of the AI tool complied with data privacy, intellectual property rights, and ethical standards.

## CRediT authorship contribution statement

**Raphael Moll:** Conceptualization, Data curation, Formal analysis, Investigation, Methodology, Supervision, Validation, Visualization, Writing – original draft, Writing – review & editing. **Tim Hoffmann:** Data curation, Methodology, Writing – review & editing. **Calvin Tu:** Data curation, Methodology, Writing – review & editing. **Roland Thünauer:** Writing – review & editing. **Wolfgang R. Streit:** Conceptualization, Formal analysis, Funding acquisition, Investigation, Resources, Supervision, Validation, Writing – original draft, Writing – review & editing. **Ifey Alio:** Conceptualization, Data curation, Formal analysis, Funding acquisition, Investigation, Methodology, Project administration, Supervision, Validation, Visualization, Writing – original draft, Writing – review & editing.

## Declaration of competing interest

The authors declare the following financial interests/personal relationships which may be considered as potential competing interests: Raphael Moll reports financial support, administrative support, article publishing charges, and equipment, drugs, or supplies were provided by University of Hamburg. Prof. Dr. Wolfgang Streit reports financial support was provided by German Research Foundation. Prof. Dr. Wolfgang Streit reports financial support was provided by Federal Ministry of Education and Research Bonn Office. If there are other authors, they declare that they have no known competing financial interests or personal relationships that could have appeared to influence the work reported in this paper.

## Data Availability

Data will be made available on request.

## Appendix A Supplementary data

Multimedia component 1 Primer list.

Multimedia component 2 Single-species biofilms (biofilm volume).

Multimedia component 3 Dual-species biofilms (biofilm volume).

Multimedia component 4 Dual-species biofilms (species distribution).

Multimedia component 5 Reporter fusions (Plate reader data).

Multimedia component 6 Reporter fusions (Heatmap).
