# Supplementary material for: Species interactions are key to spatiotemporal gene expression and multilayer formation in Stenotrophomonas maltophilia K279a dual species biofilms
Source: Biofilm. 2026 Jun 16;12:100374. doi: 10.1016/j.bioflm.2026.100374 (PMC13311295; doi:10.1016/j.bioflm.2026.100374)
Supplement: Multimedia component 7 [file mmc7.docx]

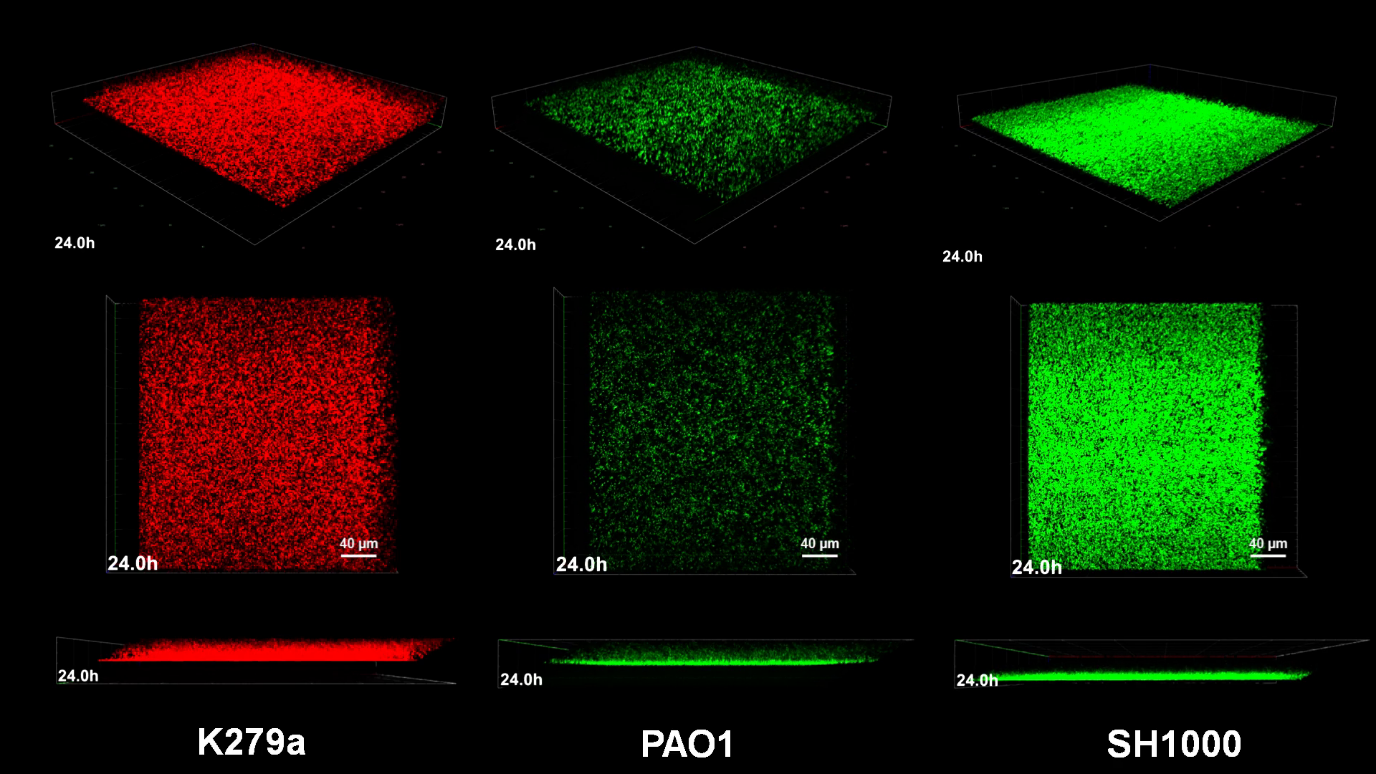


**MOVIE 1:**

**Time-lapse fluorescence microscopy reveals distinct biofilm formation dynamics of *Stenotrophomonas maltophilia* K279a, *Staphylococcus aureus* SH1000, *and Pseudomonas aeruginosa* PAO1 over 24 h.** Time-lapse fluorescence microscopy visualizing mono-species biofilm development from 0 to 24 h at 37°C under static conditions in 10% LB medium.K279a is chromosomally tagged with tdTomato and imaged in top view, side view, and 3D projection.PAO1 is chromosomally tagged with sfGFP and shown in top view, side view, and 3D projection.SH1000 expresses sfGFP from a plasmid and is presented in top view, side view and 3D projection. Microscopy was performed using a lattice light sheet microscope, enabling high-resolution, volumetric imaging of biofilm architecture with minimal phototoxicity.
The video illustrates differences in spatial biofilm architecture, biomass accumulation, and structural organization between the three strains over time.


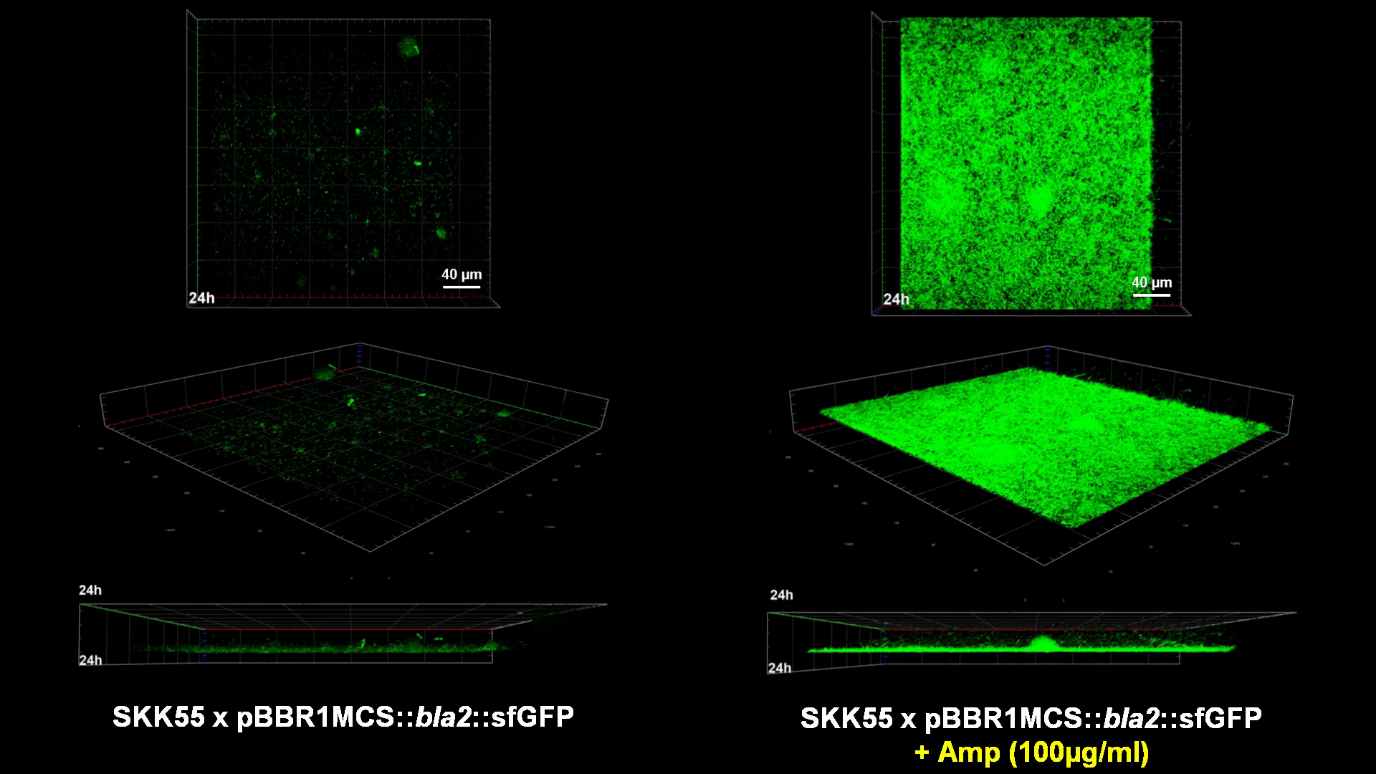


**MOVIE 2:**

**Ampicillin induces bla2 expression in *Stenotrophomonas maltophilia* SKK55 biofilms monitored by an sfGFP reporter fusion.** Time-lapse fluorescence microscopy of *S. maltophilia* SKK55 carrying the reporter construct pBBR1MCS::*bla2*-Promoter::sfGFP, imaged over 24 h under static conditions at 37°C in 10% LB medium. Microscopy was performed using a lattice light sheet microscope, enabling high-resolution, volumetric imaging of biofilm architecture with minimal phototoxicity. The left panel shows biofilm formation in the presence of Ampicillin (100 µg/ml), while the right panel displays the control without antibiotic exposure. A strong increase in sfGFP fluorescence is observed in the Ampicillin-treated condition, indicating transcriptional activation of the *bla2* promoter in response to β-lactam stress. In contrast, minimal fluorescence is detected in the control condition. This video demonstrates that Ampicillin serves as an inducer of *bla2* expression during biofilm growth, highlighting the role of antibiotic-triggered resistance mechanisms in *S. maltophilia*.


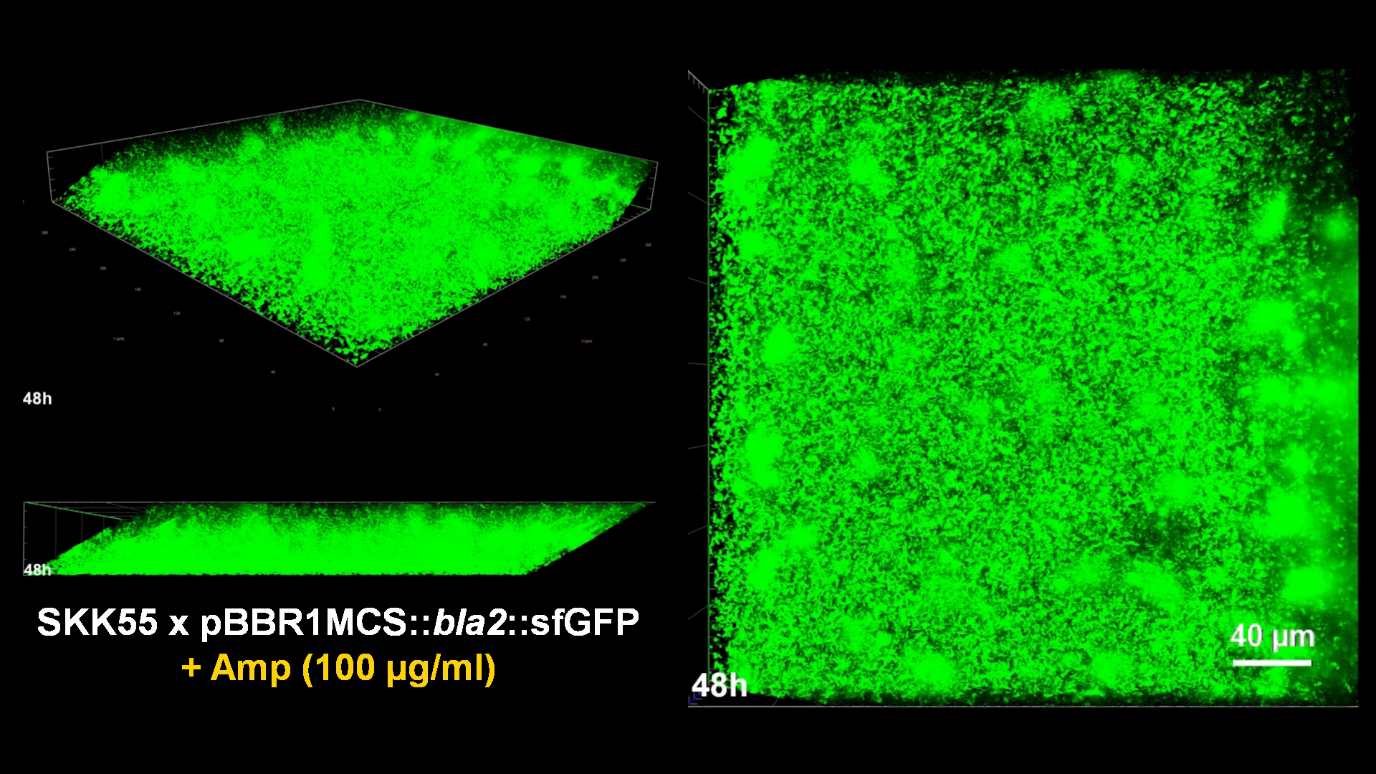


**MOVIE 3:**

**Ampicillin triggers induction of bla2 expression in *Stenotrophomonas maltophilia* SKK55 biofilms.** Time-lapse fluorescence microscopy of *S. maltophilia* SKK55 carrying the reporter construct pBBR1MCS::*bla2*-Promoter::sfGFP, recorded over 48 h under static conditions at 37°C in 10% LB medium. Microscopy was performed using a lattice light sheet microscope, enabling high-resolution, volumetric imaging of biofilm architecture with minimal phototoxicity. Ampicillin (100 µg/ml) was added after 24 h of biofilm growth. The video captures both the initial 24-h pre-treatment phase and the subsequent 24 h post-Ampicillin exposure. A marked increase in sfGFP fluorescence is observed after antibiotic addition, indicating strong activation of the *bla2* promoter. In contrast, fluorescence remains low during the initial 24 h without antibiotic, confirming that Ampicillin serves as a trigger for *bla2* expression. This video highlights the inducible nature of *bla2* in response to β-lactam stress during established biofilm growth.


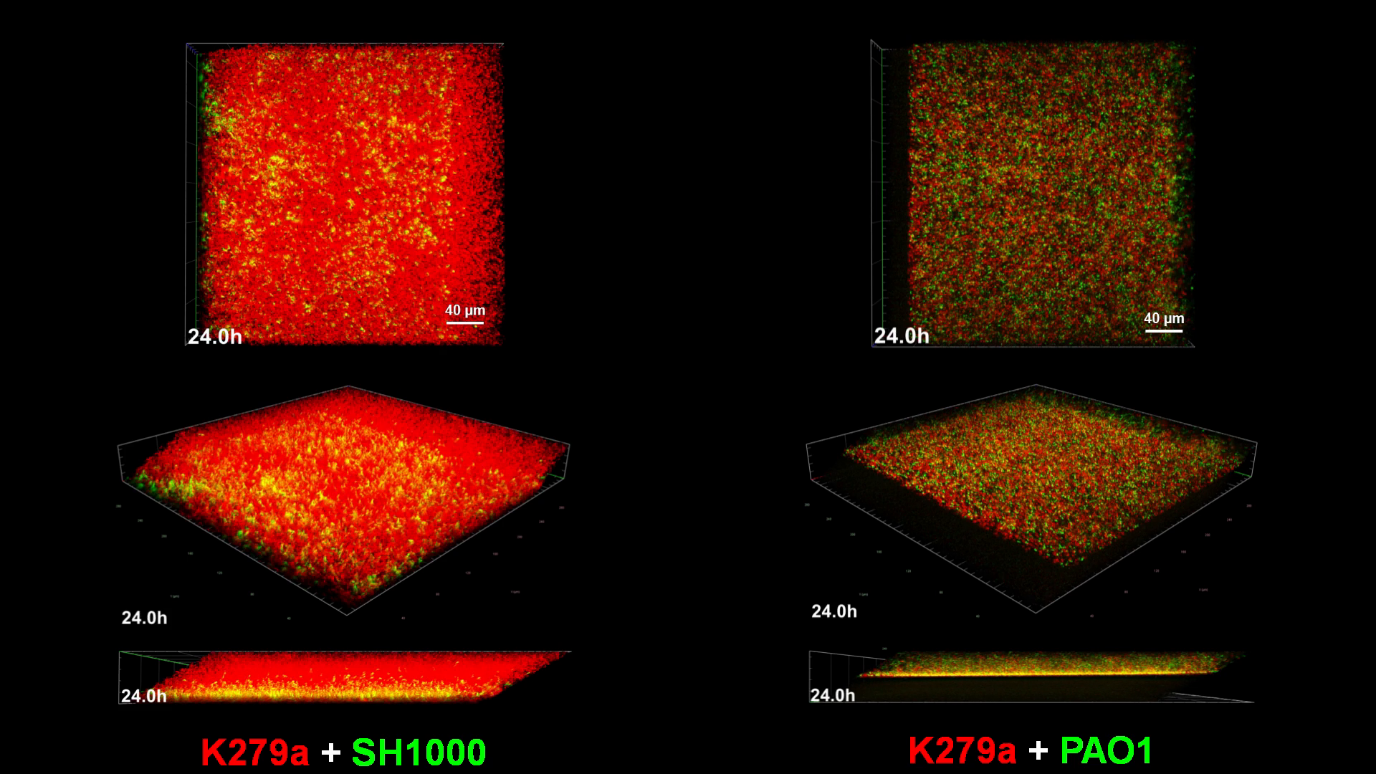


**MOVIE 4:**

***Stenotrophomonas maltophilia* K279a exhibits distinct spatial dynamics in dual-species biofilms with *Staphylococcus aureus* SH1000 and *Pseudomonas aeruginosa*** **PAO1 for over 24 h.** Time-lapse fluorescence microscopy visualizing dual-species biofilm development from 0 to 24 h at 37°C under static conditions in 10% LB medium.
*K279a* is chromosomally tagged with tdTomato (red). *SH1000* expresses sfGFP from a plasmid, and *PAO1* is chromosomally tagged with sfGFP (both shown in green). Each co-culture is presented using fluorescence channel overlays to highlight spatial distribution and structural dynamics over time. Microscopy was performed using a lattice light sheet microscope, enabling high-resolution, volumetric imaging of biofilm architecture with minimal phototoxicity. The video reveals distinct interactions between *K279a* and each partner strain, with differences in spatial organization, surface colonization, and competitive or cooperative biofilm development.


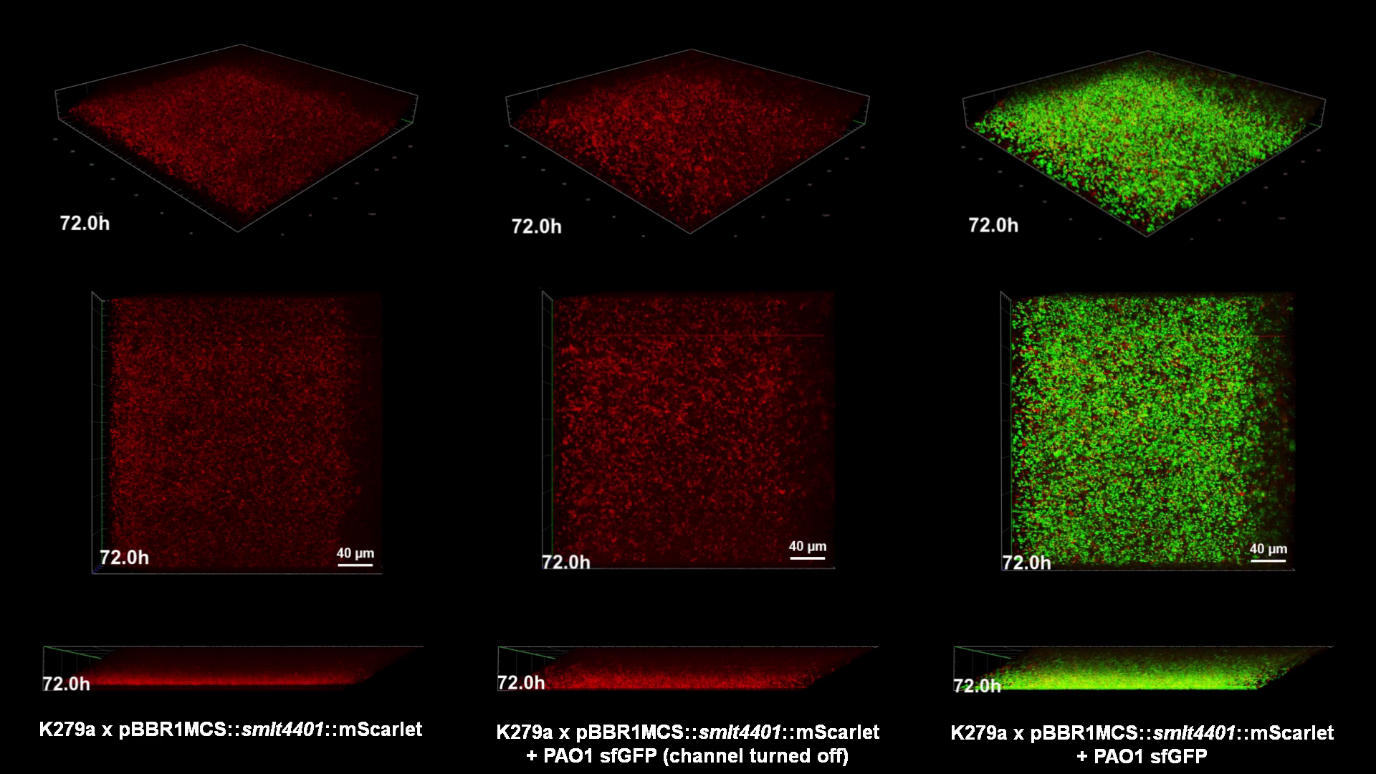


**MOVIE 5:**

**Cocultivation with *Pseudomonas aeruginosa* PAO1 induces smlt4401 reporter expression in *Stenotrophomonas maltophilia* K279a biofilms.** The biofilms were visualized from multiple angles using confocal laser scanning microscopy. **Left:** Biofilm of *S. maltophilia* K279a expressing the reporter construct grown in monoculture. **Middle:** Coculture biofilm of reporter-expressing *S. maltophilia* K279a with *Pseudomonas aeruginosa* PAO1 expressing sfGFP. **Right:** Same coculture as in the middle panel, with the green fluorescence channel (sfGFP) turned off to enhance visualization of *smlt**4401*::mScarlet expression. Biofilms were grown in 10% LB at 37°C under static conditions. Increased red fluorescence signal in the presence of PAO1 indicates induction of *smlt4401* expression by cocultivation.


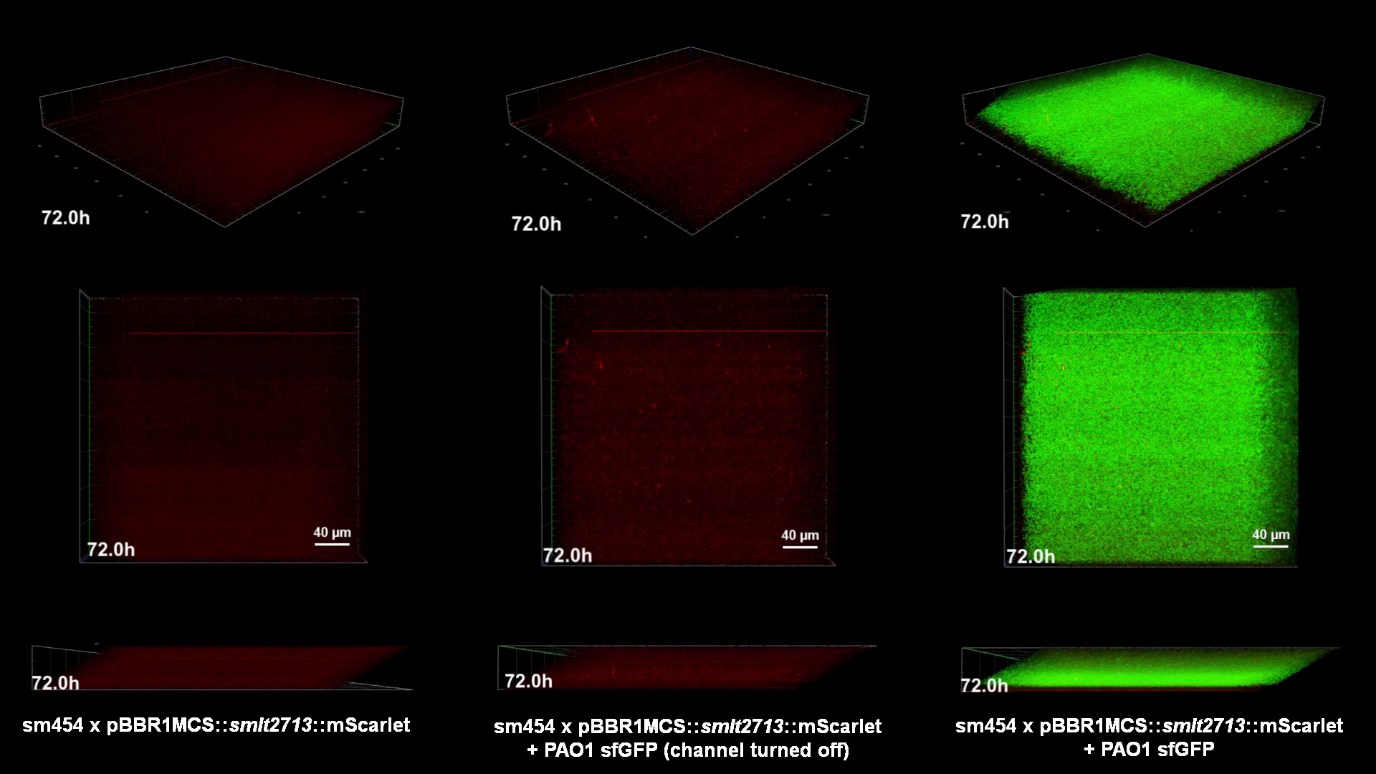


**MOVIE 6:**

**Cocultivation with *Pseudomonas aeruginosa* PAO1 induces smlt2713 reporter expression in *Stenotrophomonas maltophilia* sm454 biofilms.** The biofilms were visualized from multiple angles using confocal laser scanning microscopy. **Left:** Biofilm of *S. maltophilia* sm454 expressing the reporter construct grown in monoculture. **Middle:** Coculture biofilm of reporter-expressing *S. maltophilia* sm454 with *Pseudomonas aeruginosa* PAO1 expressing sfGFP. **Right:** Same coculture as in the middle panel, with the green fluorescence channel (sfGFP) turned off to enhance visualization of *smlt**2713*::mScarlet expression. Biofilms were grown in 10% LB at 37°C under static conditions. Increased red fluorescence signal in the presence of PAO1 indicates induction of *smlt2713* expression by cocultivation.


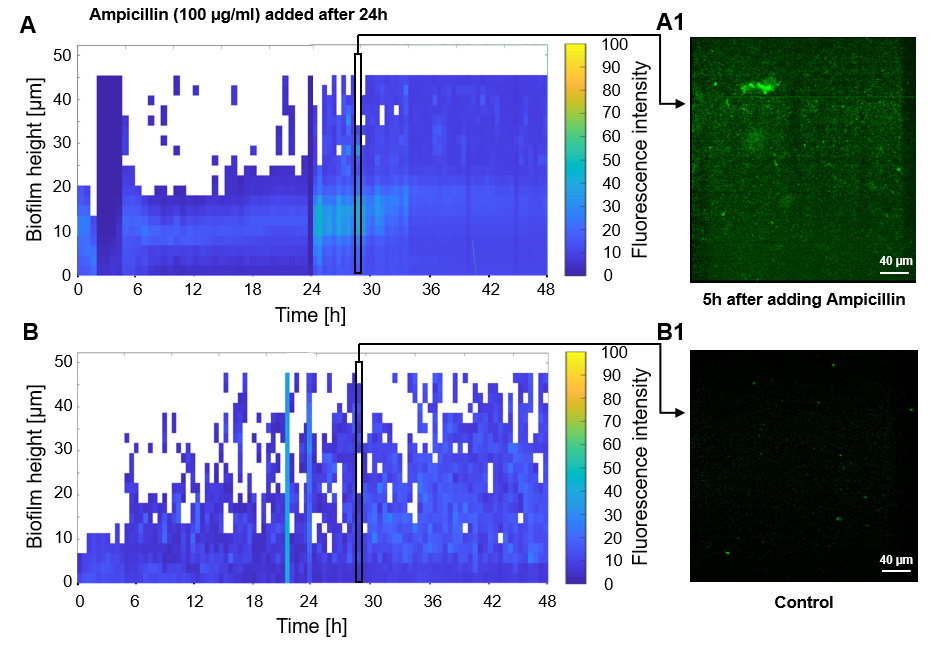


**FIGURE S1: Biofilm formation and fluorescence intensity in SKK55 are differentially regulated by Ampicillin-induced Bla2 reporter expression. (A)** Heatmap depicting biofilm height and fluorescence intensity of SKK55 harboring the reporter fusion pBBR1MCS::*bla2*::sfGFP after 24 h of growth. Following this period, Ampicillin (100 µg/ml) was introduced, triggering the expression of the reporter fusion. (**A1)** Confocal laser scanning image illustrating the biofilm structure under static conditions at 37°C in 10% LB medium, taken 5 h post-Ampicillin addition, demonstrating enhanced fluorescence indicative of reporter expression. **(B)** Control heatmap showing biofilm height and fluorescence intensity of SKK55 with the same reporter fusion pBBR1MCS::*bla2*::sfGFP, but without the addition of Ampicillin. (**B1)** Confocal laser scanning image of the biofilm grown under static conditions at 37°C in 10% LB medium for 29 h, revealing the biofilm's structural characteristics in the absence of Ampicillin-induced reporter expression. Microscopy was performed using a lattice light sheet microscope, enabling high-resolution, volumetric imaging of biofilm architecture with minimal phototoxicity. Quantification was performed using the software BiofilmQ. Overall, the heatmaps and confocal images illustrate the differential effects of Ampicillin on biofilm formation and fluorescence intensity in SKK55, highlighting the role of antibiotic-induced gene expression mediated by Bla2 in biofilm dynamics and the organism's resilience against antibiotic treatment.


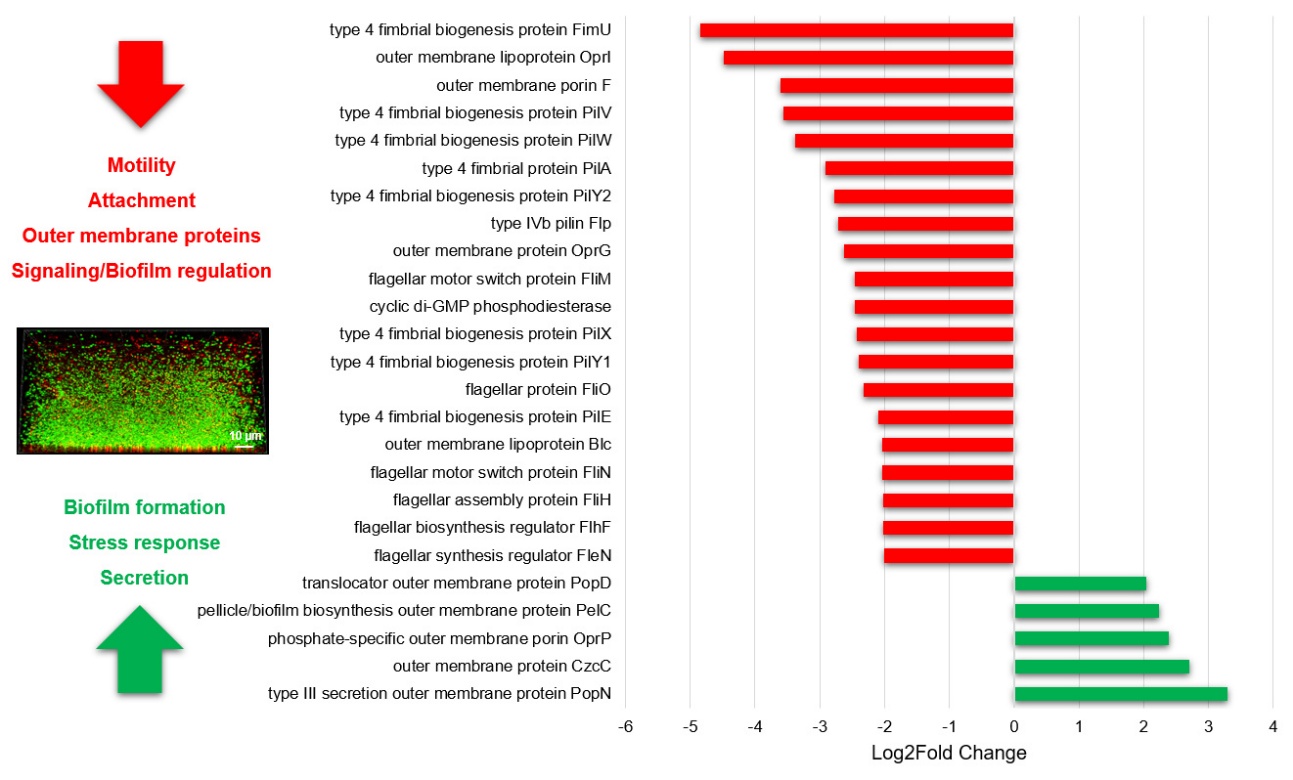


**FIGURE S2:**

***Stenotrophomonas maltophilia* K279a modulates attachment- and motility-associated gene expression in *Pseudomonas aeruginosa* PAO1 during dual-species biofilm growth.** Transcriptomic analysis of *P. aeruginosa* PAO1 genes from 72-h static co-culture biofilms with *S. maltophilia* K279a grown at 37°C in 10% LB. The bar plot shows the Log2Fold Change of PAO1 genes associated with motility, attachment, outer membrane components, signaling, and biofilm regulation. Red bars indicate significant downregulation of genes involved in type IV fimbriae biogenesis, flagellar assembly, and cyclic di-GMP signaling, all critical for initial surface attachment and motility. In contrast, green bars highlight upregulated genes related to biofilm maturation, stress adaptation, and secretion. The overall gene expression profile suggests that *S. maltophilia* K279a outcompetes PAO1 in early surface colonization and likely suppresses PAO1’s attachment capacity, leading to reduced motility and altered biofilm behavior in the dual-species setting.


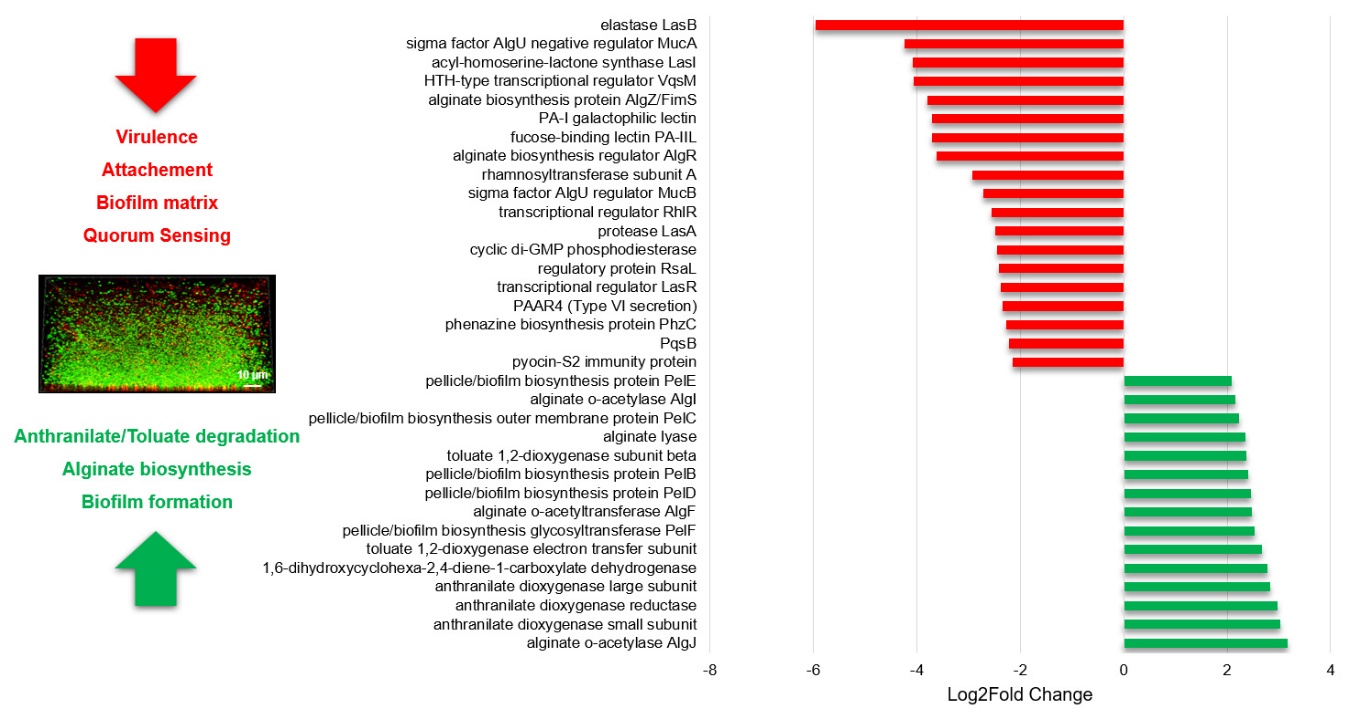


**FIGURE S3:**

**Dual-species biofilm growth with *S. maltophilia* K279a modulates Quorum Sensing and QS-linked genes in *P. aeruginosa* PAO1.** Transcriptomic analysis was performed on 72-hour static co-culture biofilms grown at 37°C in 10% LB. The bar graph shows significantly downregulated Quorum Sensing (QS) and QS-associated genes (e.g., *lasA, lasB, lasI, lasR, rhlR, vqsM*), expressed aslog2 fold change, in PAO1 during co-culture compared to single-species biofilms, indicating suppression of major QS regulatory pathways and virulence factors. Conversely, several genes involved in alginate modification, pellicle/biofilm biosynthesis, and aromatic compound metabolism, which are also linked to QS regulation, were upregulated. This suggests that while key QS regulatory pathways are suppressed, specific QS-associated genes related to biofilm matrix production and secondary metabolism may be selectively activated during dual-species biofilm growth.


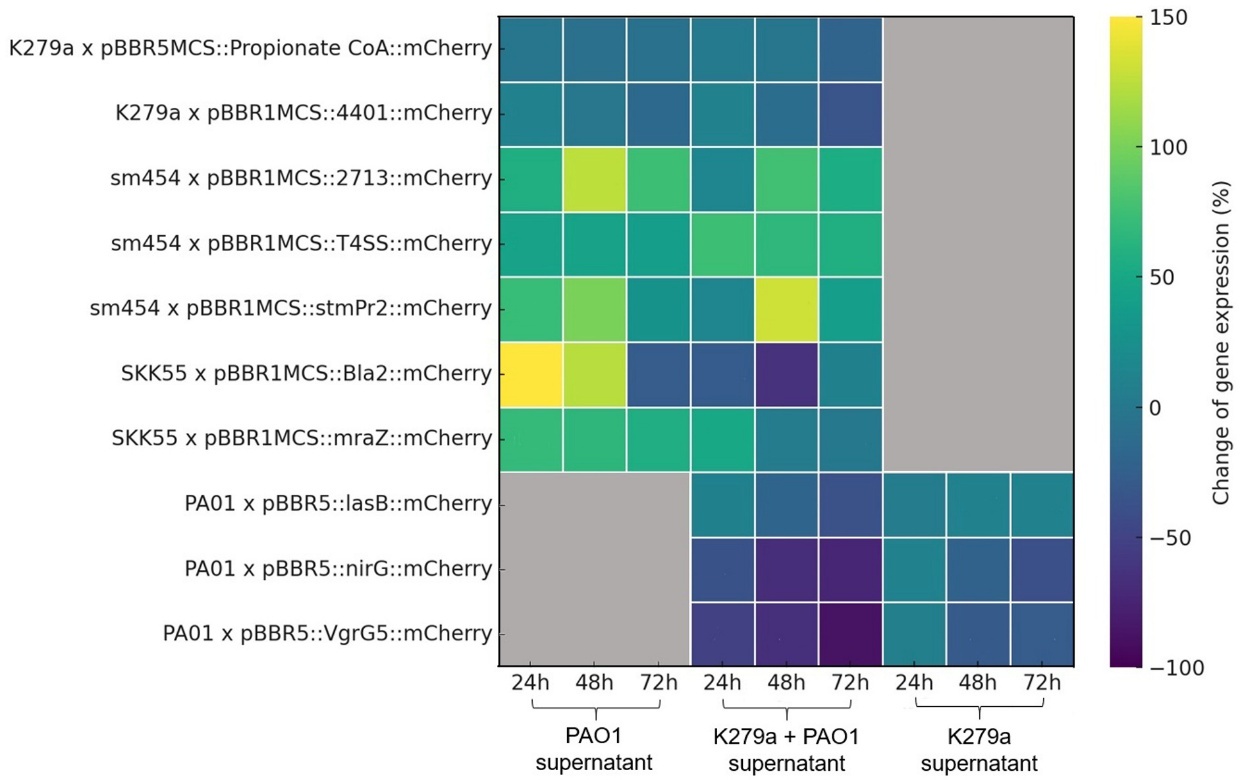


**Figure S4: Exposure to biofilm supernatants modulates the expression of stress and virulence-associated genes in reporter strains.** Fluorescence intensities of *Stenotrophomonas maltophilia* K279a and *Pseudomonas aeruginosa* PAO1 reporter strains carrying mCherry transcriptional fusions were measured over time after exposure to biofilm-derived supernatants. Each row represents a different promoter::mCherry construct, and each column indicates the condition (type of supernatant) and time point (24h, 48h, 72h). The supernatants used were from K279a, PAO1 and a 1:1 mix of both. Cells were cultured in 10% LB and exposed to the respective supernatants in 96-well microtiter plates under static conditions at 37°C for 24 h. Fluorescence was normalized to OD₆₀₀ to account for differences in cell density. Values represent the percentage change in gene expression relative to the untreated control for each construct and time point. Color scale indicates the magnitude and direction of change in gene expression (increase in yellow, decrease in purple, unchanged in green/blue). Gray boxes denote not available data. Three independent biological replicates with six technical replicates each were performed.


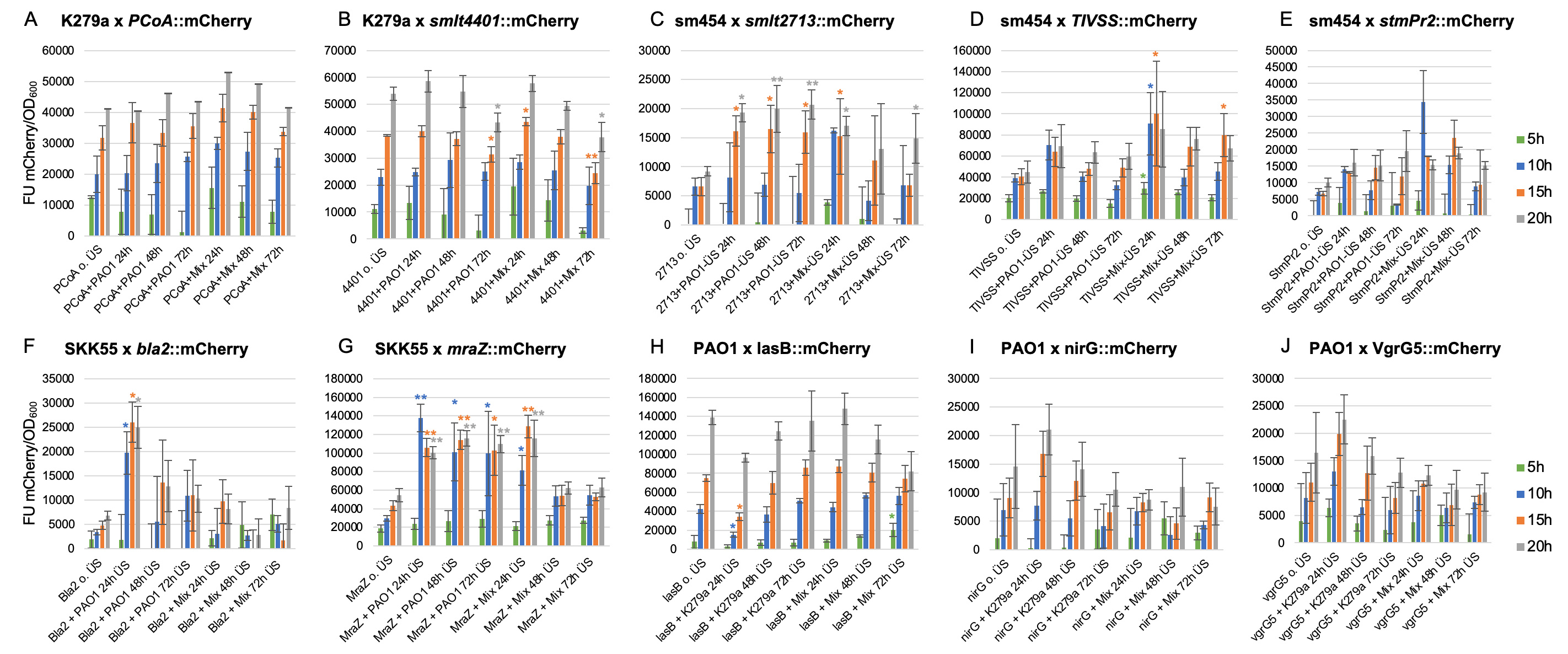
**FIGURE S5: Reporter gene activity is differentially regulated by supernatants from single-species and mixed-species biofilms.** Reporter strains were incubated with sterile-filtered biofilm supernatants collected after 24 h, 48 h, and 72 h of biofilm growth from K279a, PAO1, or mixed K279a + PAO1 biofilms to determine whether extracellular factors influence reporter gene expression. The following reporter strains were tested: **(A)** K279a x *PCoA*::mCherry*,* **(B)** K279a x *smlt4401*::mCherry*,* **(C)** sm454 x *smlt2713*::mCherry*,* **(D)** sm454 x *TIVSS*::mCherry*,* **(E)** sm454 x *stmPr2*::mCherry*,* **(F)** SKK55 x *bla2*::mCherry*,* **(G)** SKK55 x *mraZ*::mCherry*,* **(H)** PAO1 x *lasB*::mCherry*,* **(I)** PAO1 x *nirG*::mCherry*,* **(J)** PAO1 x *vgrG5*::mCherry. Reporter strains derived from K279a, sm454, and SKK55 were treated with supernatants from PAO1 and mixed biofilms K279a+PAO1. Reporter strains derived from PAO1 were treated with supernatants from K279a and mixed biofilms K279a+PAO1. Untreated reporter cultures served as controls. Reporter biofilms were grown in 10% LB at 37°C for 24 h under static conditions in 96-well plates. For each condition, mCherry fluorescence was measured and normalized to OD600 after 5 h (green), 10 h (blue), 15 h (orange), and 20 h (grey) of biofilm growth. OD600 and fluorescence intensity were measured using an Infinite® 200 PRO plate reader (Tecan). Data are presented as mean fluorescence intensity ± standard deviation from three independent experiments, each with three technical replicates. Statistical analyses were performed by comparing each treatment condition to the untreated control (no supernatant). Statistical significance is indicated as follows: p ≥ 0.05, not significant (no asterisk); p < 0.05, *; p < 0.01, ****.**


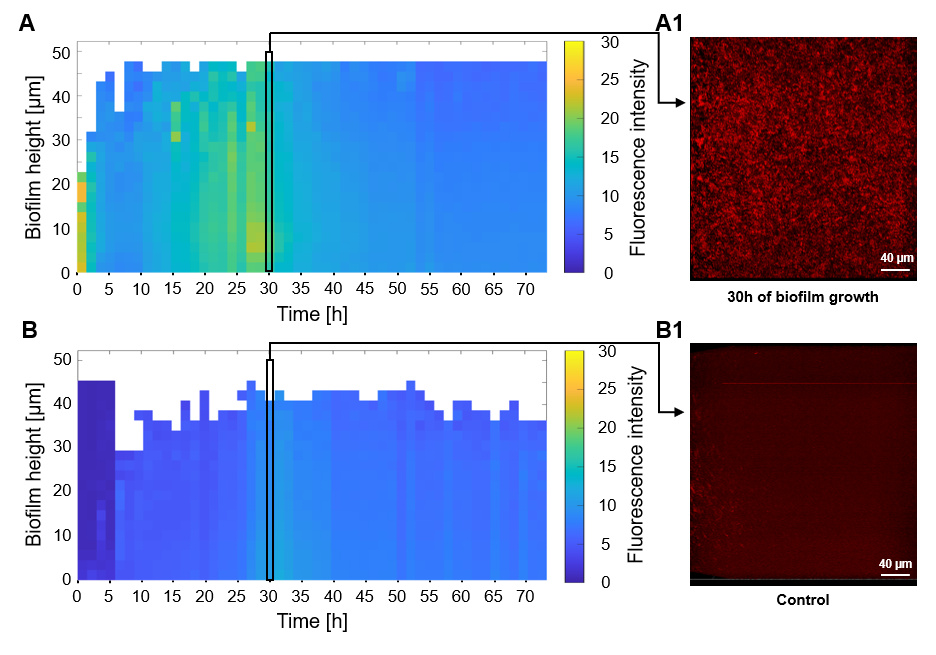


**FIGURE S6: Coculture with *Pseudomonas aeruginosa* PAO1 modulates biofilm structure and respiration-associated gene expression in *Stenotrophomonas maltophilia* K279a. (A)** Heatmap depicting biofilm height and fluorescence intensity of K279a harboring the reporter fusion pBBR1MCS::*smlt4401*::mScarlet which was cocultivated with PAO1 sfGFP between 0-72 h of growth. (**A1)** Confocal laser scanning image illustrating the biofilm structure under static conditions at 37°C in 10% LB medium, taken after 30 h of growth, demonstrating enhanced fluorescence indicative of reporter expression. **(B)** Control heatmap showing biofilm height and fluorescence intensity of K279a with the same reporter fusion pBBR1MCS::*smlt4401*::mScarlet, but without the addition of PAO1 sfGFP. (**B1)** Confocal laser scanning image of the biofilm grown under static conditions at 37°C in 10% LB medium for 30 h, revealing the biofilm's structural characteristics in the absence of PAO1 sfGFP. Microscopy was performed using a lattice light sheet microscope, enabling high-resolution, volumetric imaging of biofilm architecture with minimal phototoxicity. Quantification was performed using the software BiofilmQ. Overall, the heatmaps and confocal images illustrate the differential effects of PAO1 on biofilm formation and fluorescence intensity in K279a, highlighting the role of PAO1 induced gene expression mediated by smlt4401 in biofilm respiration.


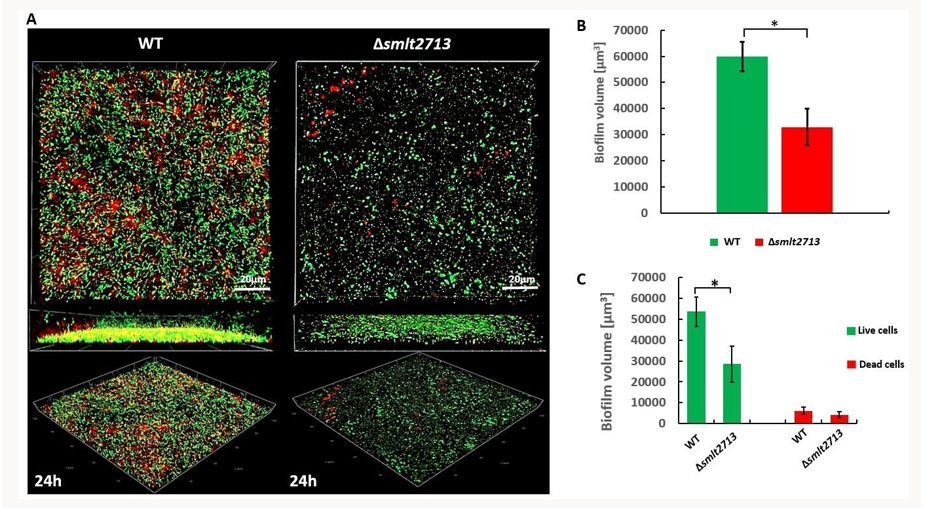


**FIGURE S7: *Stenotrophomonas maltophilia* sm454 Δ*smlt2713* shows impaired biofilm formation under EDTA-supplemented conditions.** Confocal laser scanning microscopy images illustrating the biofilm architecture of *S. maltophilia* sm454 wild type and the Δ*smlt2713* mutant after **24 hours** of growth under static conditions in **10% LB supplemented with 1 mM EDTA at 37°C**. A LIVE/DEAD staining was performed before imaging. The biofilms images highlight pronounced structural differences between both strains, with the wild type forming a dense and spatially organized biofilm, whereas the Δ*smlt2713* mutant displays reduced growth and lacks a clearly developed biofilm structure. **(B)** Quantification of total biofilm volume using the software **BiofilmQ** demonstrates a significant reduction in biofilm biomass of the Δ*smlt2713* mutant compared to the wild type after 24 hours of incubation. **(C)** Separate quantification of live and dead cell populations within the biofilms shows that the reduced biofilm volume is mainly associated with the live cell fraction. A significant difference between wild type and Δ*smlt2713* was observed for live cells, whereas no significant difference was detected for dead cells. Statistical analysis was performed using a **Student’s t-test**. Error bars indicate **standard deviation**. Asterisks indicate statistically significant differences with **p < 0.05**. Together, these results demonstrate that *smlt2713* contributes to robust biofilm formation of *S. maltophilia* sm454 under EDTA-supplemented conditions and that loss of this gene impairs biofilm development and biomass accumulation.


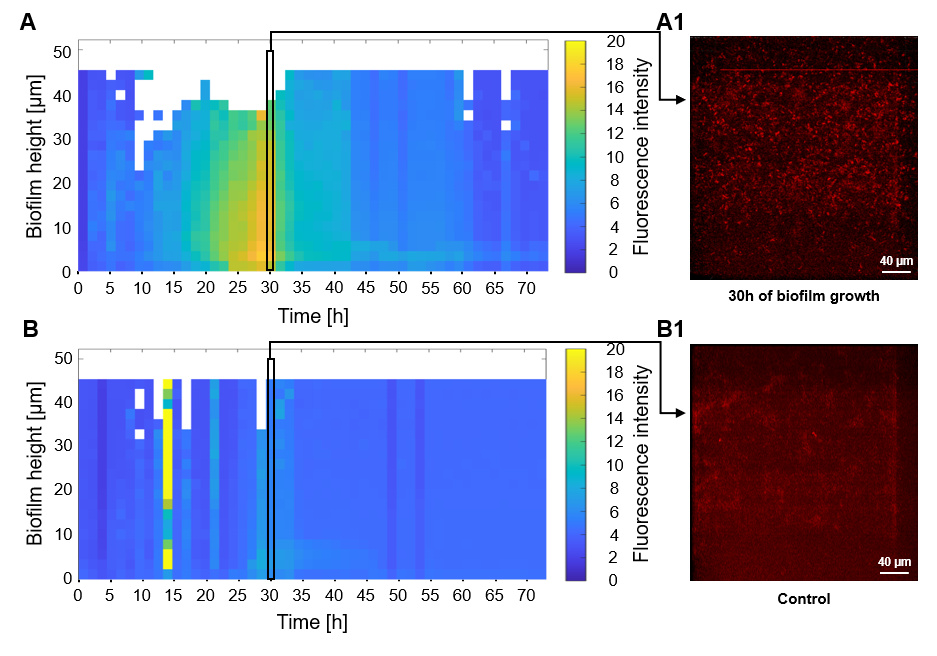


**FIGURE S8: Cocultivation with *Pseudomonas aeruginosa* PAO1 induces smlt2713 reporter expression and modulates biofilm formation in *Stenotrophomonas maltophilia* sm454. (A)** Heatmap depicting biofilm height and fluorescence intensity of sm454 harboring the reporter fusion pBBR1MCS::*smlt2713*::mScarlet which was cocultivated with PAO1 sfGFP between 0-72 h of growth. (**A1)** Confocal laser scanning image illustrating the biofilm structure under static conditions at 37°C in 10% LB medium, taken after 30 h of growth, demonstrating enhanced fluorescence indicative of reporter expression. **(B)** Control heatmap showing biofilm height and fluorescence intensity of sm454 with the same reporter fusion pBBR1MCS::*smlt2713*::mScarlet, but without the addition of PAO1 sfGFP. (**B1)** Confocal laser scanning image of the biofilm grown under static conditions at 37°C in 10% LB medium for 30 h, revealing the biofilm's structural characteristics in the absence of PAO1 sfGFP. Microscopy was performed using a lattice light sheet microscope, enabling high-resolution, volumetric imaging of biofilm architecture with minimal phototoxicity. Quantification was performed using the software BiofilmQ. Overall, the heatmaps and confocal images illustrate the differential effects of PAO1 on biofilm formation and fluorescence intensity in sm454, highlighting the role of PAO1 induced gene expression mediated by smlt2713.
